# A Leakage-Controlled Benchmark of Metaheuristic Multi-Omics Feature Selection for Reconstructing TCGA Molecular Subtypes in Endometrial Cancer

**DOI:** 10.3390/cancers18203353

**Published:** 2026-10-16

**Authors:** Minyue Cao, Yiqin Zhang, Yan Ding, Genyi Jiang, Jiayu Yan, Yihan Sun, Yanli Zhang, Luyao Kang, Jing Luo, Bilan Li

**Affiliations:** Shanghai Key Laboratory of Maternal Fetal Medicine, Shanghai Institute of Maternal-Fetal Medicine and Gynecologic Oncology, Shanghai First Maternity and Infant Hospital, School of Medicine, Tongji University, Shanghai 200092, China; minlei0513@163.com (M.C.); cathefrine811@163.com (Y.Z.); rdingyan@163.com (Y.D.); jianggenyi@163.com (G.J.); yanjiayu@51mch.com (J.Y.); sunyh@tongji.edu.cn (Y.S.); zhangyanlia@51mch.com (Y.Z.); luyaokang@tongji.edu.cn (L.K.); 2532243@tongji.edu.cn (J.L.)

**Keywords:** endometrial carcinoma, molecular subtyping, multi-omics data integration, feature selection, metaheuristic optimization

## Abstract

**Background:** The four molecular subtypes of endometrial carcinoma defined by The Cancer Genome Atlas (TCGA) can be reconstructed from multi-omics data, but these data contain about 460,000 features for 369 patients, the subtypes are imbalanced, and feature selection easily leaks test-set information. **Methods:** We developed POISE-X, a leakage-controlled, imbalance-aware benchmarking pipeline with an interchangeable metaheuristic search engine. Using RNA-seq, miRNA, DNA methylation, copy-number, mutation, and clinical data from 369 TCGA-UCEC patients, we compared 19 optimizers under nested five-fold cross-validation, with in-fold prefiltering to 2505 candidate features and a macro-averaged $F_{1}$ fitness function. **Results:** Performance differed across optimizers in a Friedman test on fold means ($p=1.989\times 10^{-6}$). No selected panel exceeded logistic regression on the full candidate pool, which reached a macro-averaged $F_{1}$ of 0.902; the compact configuration chosen by our selection rule, POISE-GWO, retained 443 features with a macro-averaged $F_{1}$ of 0.880, and the selection rule itself scored 0.881 under leave-one-fold-out evaluation, and an ANOVA-ranked panel of 450 features reached 0.886. These results held without clinical variables and, in the two searches tested, for parsimony weights of 0.8–0.99. A surrogate of the clinical molecular classification built from *POLE* exonuclease-domain mutation, mismatch-repair deficiency, and *TP53* mutation reached a macro-averaged $F_{1}$ of 0.924, higher on average than every multi-omics model. **Conclusions:** In this cohort, wrapper search therefore compressed the feature set without adding discriminative power, and the selected panels reconstructed an existing classification, not a new treatment-relevant signature. POISE-X is a framework for benchmarking feature-selection strategies; any selected panel requires external validation before clinical use.

## Simple Summary

Endometrial cancer can be divided into four molecular subtypes that help predict outcome and guide treatment. These subtypes can be inferred from genomic data, but such data contain hundreds of thousands of measurements for only a few hundred patients, which makes it easy to overestimate how well a model works. We built POISE-X, a carefully controlled testing framework, and we used it to compare 19 search algorithms that chose a small set of informative measurements from the data of 369 patients. The algorithms reduced the number of measurements needed, but none classified the subtypes better than a simple model using all candidate measurements. A three-marker rule based on the classification used in the clinic (*POLE* mutation, mismatch-repair deficiency, and *TP53* mutation) had the highest average score of all. POISE-X therefore helps researchers compare feature-selection methods fairly, whereas the selected marker sets are not ready for clinical use.

## 1. Introduction

Endometrial carcinoma is the most common gynecological malignancy in high-income countries, and its clinical management is increasingly guided by molecular criteria in addition to histology. The Cancer Genome Atlas (TCGA) integrated analysis of uterine corpus endometrial carcinoma (UCEC) defined four molecular subtypes with distinct genomic drivers and distinct prognoses: *POLE*-ultramutated tumors, microsatellite-instability (MSI) hypermutated tumors, copy-number-low (endometrioid-like) tumors, and copy-number-high (serous-like, *TP53*-mutated) tumors [1]. Surrogate classifiers such as ProMisE reproduce these groups with a small panel of clinically deployable assays [2,3], and the 2021 joint guidelines of the European Society of Gynaecological Oncology, the European Society for Radiotherapy and Oncology, and the European Society of Pathology (ESGO/ESTRO/ESP) recommend molecular classification for risk stratification and adjuvant-treatment decisions [4]. Identifying the subtype of a tumor is therefore a prediction task with direct therapeutic consequences: patients with *POLE*-mutated tumors have an excellent prognosis and may be spared adjuvant therapy, whereas copy-number-high tumors warrant the most intensive treatment. In routine care, the subtype is assigned by these clinical assays. The question addressed in this study is a different, methodological one: how well, and with how many features, high-dimensional multi-omics data reconstruct the existing TCGA classification, and whether metaheuristic feature selection adds to simple baselines in doing so. Because the TCGA classes are themselves defined by genomic properties, the answer measures the reconstruction of a known classification, not the discovery of a new treatment-relevant signature.

Multi-omics profiling makes this prediction possible from data alone, but it poses a severe dimensionality problem. For the TCGA-UCEC cohort used in this work, five omics layers (i.e., RNA-seq gene expression, miRNA expression, 450K DNA methylation, gene-level copy number, and somatic mutations) together with structured clinical variables amount to 459,471 measured features for 369 patients after quality control. Learning a classifier directly in this space is statistically fragile and clinically uninformative, because it cannot indicate which molecular measurements carry the subtype signal. Feature selection is the natural remedy: it reduces the input to a compact panel and, if done carefully, can improve generalization [5]; whether it does so on a given problem has to be tested against simple baselines. Among feature-selection strategies, wrapper methods that score candidate subsets by the cross-validated performance of a downstream classifier are the most directly aligned with the prediction goal [6], and metaheuristic optimizers—evolutionary, swarm-based, and physics-inspired algorithms—are the standard way of searching the combinatorial space of subsets that wrappers create. A recent review of 91 studies finds particle swarm optimization, genetic algorithms, and ant colony optimization to be the most frequently used metaheuristics for feature selection and network optimization in cancer classification [7], and metaheuristic feature selectors have been applied to gene expression [8,9,10], multi-omics [11,12], and histopathology [13,14] data.

Two properties of the molecular-subtyping task are, however, routinely under-addressed in this literature. The first is class imbalance. In the cohort analyzed here the four subtypes accounted for 10%, 28%, 28%, and 33% of patients, and the rarest class, *POLE*-ultramutated, was precisely the one whose recognition changed clinical management the most. A wrapper whose fitness function rewards overall accuracy—the choice made, for example, in the Harris hawks wrapper of Manhrawy et al. [8]—can reach a high score while sacrificing the minority class entirely, because misclassifying every *POLE* tumor costs only about ten percentage points of accuracy. The asymmetry of the label distribution therefore propagates into the search objective and, from there, into the selected features. The second property is evaluation leakage. Univariate prefiltering and the wrapper search itself both consume the class labels; if either of them is performed on the full cohort before cross-validation then the reported performance is optimistically biased [15]; feature standardization uses no labels but is also fitted on the training rows only in our pipeline. Both issues are well known in principle, yet they are rarely handled jointly and explicitly in metaheuristic feature-selection studies on cancer omics data.

A third consideration shaped the design of the pipeline. Of all its components, the wrapper search is the only one whose choice is not dictated by the task itself. The no-free-lunch theorems imply that no search algorithm is uniformly superior [16], and the past decade has produced a large number of nature-inspired optimizers whose relative merit on a given problem class can only be established empirically. Application papers typically settle this choice by convention, adopting one optimizer and comparing it against three or four familiar baselines. We instead designed the search engine as a plug-and-play component: any population-based optimizer that operates on a continuous, box-constrained search space can be inserted without changing the encoding, the fitness function, the classifier pool, or the evaluation protocol. This makes the choice of optimizer an empirical question that the pipeline itself can address, within the resolution that the cohort allows. We instantiate the component with 19 metaheuristics available in the MEALPY library [17], evaluate all of them under identical conditions, and we report the comparison as a benchmark. To make the choice of a single compact configuration transparent, we apply an explicit rule: among the instantiations whose classification performance is not significantly different from the best, we choose the one whose searches reach the lowest value of the objective; we then evaluate this rule itself by leave-one-fold-out selection.

This paper presents a Plug-and-play Optimizer for Imbalance-aware Subtyping of Endometrial cancer (POISE-X), a class-imbalance-aware multi-omics feature-selection pipeline for endometrial cancer molecular subtyping with an interchangeable metaheuristic search engine. The name reflects the two ideas that the pipeline rests on: the placeholder X stands for the search engine, a component that can be exchanged without touching the rest of the pipeline, and the objective holds the four subtypes in balance regardless of their prevalence. The proposed POISE-X (i) integrates five omics layers and clinical variables of the TCGA-UCEC cohort into a single patient-by-feature matrix; (ii) performs label-dependent prefiltering, standardization, and search strictly inside the training fold of a nested cross-validation scheme, so that the outer test fold is never seen by prefiltering, standardization, or the wrapper search; (iii) searches a combined continuous space of N+5 dimensions in which the first *N* coordinates select features and the last five select which of five candidate classifiers—random forest (RF), gradient-boosted trees (XGBoost), support-vector machine (SVM), logistic regression (LR), and *k*-nearest neighbors (KNN)—form a soft-voting ensemble; (iv) drives the search with a fitness function in which the classification term is the macro-averaged $F_{1}$ score, so that each subtype contributes equally to the objective regardless of its prevalence; and (v) accepts any metaheuristic optimizer that operates on a continuous search space as its search engine. We investigated 19 candidate optimizers in this role, each run 20 times with paired random seeds under the same evaluation budget; an instantiation was named by substituting the optimizer for X, for example POISE-DE for differential evolution. The rule above designates POISE-GWO, which uses the gray wolf optimizer; we report it as one performance–compression trade-off within this benchmark, not as a validated final model. Because the candidates were compared on the same outer folds, the complete comparison is reported, not only the winner, so that the selection is transparent and the performance of the chosen configuration can be read alongside that of its alternatives.

The main contributions are as follows:

1. We implement POISE-X, a reproducible benchmarking pipeline for multi-omics feature selection and reconstruction of the TCGA molecular subtypes on TCGA-UCEC: a nested cross-validation pipeline in which prefiltering, standardization, feature selection, and classifier choice are performed separately within each outer training fold of a fixed, stratified five-fold partition, and in which the metaheuristic search engine is an interchangeable component.
2. We formulate a class-imbalance-aware wrapper objective that combines the macro-averaged $F_{1}$ score with a feature-count penalty, and we introduce a combined feature-and-classifier encoding that lets the search choose the classifier ensemble as part of the solution.
3. We systematically investigated 19 optimizers from the MEALPY library as instantiations of the POISE-X search engine—four whose wrapper applications to cancer omics data are reviewed in Section 2.3 (i.e., Harris hawks optimization, gray wolf optimizer, cuckoo search, and ant colony optimization) and 15 further evolutionary, swarm-based, physics-based, and mathematics-based algorithms, some of which, such as particle swarm optimization and differential evolution, have also been applied to cancer data—under an identical evaluation budget with non-parametric statistical testing, and we evaluated by leave-one-fold-out selection the rule used to single out one compact configuration, POISE-GWO.
4. We quantify what the wrapper search achieved relative to a no-selection reference (all prefiltered features fed to each classifier) and to an ANOVA filter of matched size, we report a stability analysis and a consensus-panel experiment that characterized how the subtype signal is distributed over the candidate features, and we tested the robustness of the results without clinical variables, over the data layers, over the parsimony weight, and against a surrogate of the clinical molecular classification. The wrapper search achieved substantial compression at a small cost in accuracy: POISE-GWO retained 443 of the 2505 candidate features, or 18%, with a macro-averaged $F_{1}$ of 0.880, close to the 0.902 of the full-pool model, and a matched-size ANOVA filter reached a comparable 0.886.

The remainder of the paper is organized as follows. Section 2 reviews related work on molecular subtyping, metaheuristic feature selection in cancer genomics, the design of wrapper objective functions, and class imbalance. Section 3 describes the data, the POISE-X pipeline, and the experimental protocol. Section 4 reports the comparison of the candidate optimizers, the compact configuration POISE-GWO and the evaluation of the rule that selects it, the no-selection reference, the stability analysis, and the sensitivity analyses. Section 5 discusses the findings and their limitations, and Section 6 concludes this paper.

## 2. Related Work

### 2.1. Molecular Subtyping of Endometrial Carcinoma

The TCGA study of 373 endometrial carcinomas established the four-class molecular taxonomy by integrating exome sequencing, copy-number arrays, RNA and miRNA sequencing, DNA methylation arrays, and proteomics [1]. Each class is defined by a characteristic genomic lesion: exonuclease-domain mutations of *POLE* with an ultra-high mutation burden; microsatellite instability, most often through *MLH1* promoter hypermethylation; a near-diploid genome with frequent *PTEN*, *CTNNB1*, *PIK3CA*, and *ARID1A* mutations; and extensive somatic copy-number alteration with *TP53* mutation. Because the original assignment required sequencing that is impractical in routine care, surrogate classifiers were developed. ProMisE reproduces the TCGA groups with *POLE* sequencing, mismatch-repair immunohistochemistry, and p53 immunohistochemistry, and it has been validated in an independent cohort [2] and in a large population-based series [3]. The 2021 European guidelines incorporate this classification into risk groups that guide adjuvant treatment [4]. These works established the subtype as a clinically actionable label; they did not, however, ask which molecular features beyond the defining lesions carry subtype information, which is the question a data-driven feature-selection study can address.

### 2.2. Machine Learning on TCGA-UCEC and Multi-Omics Feature Selection

Machine-learning studies of TCGA-UCEC have mostly used a single data modality or a single prediction target. Gargya and Bálint trained a classifier on RNA-seq data to separate grade 1 from grade 3 endometrioid tumors and, after iterative retraining, reduced it to a twelve-transcript model that stratifies grade 2 patients by relapse risk [18]. Hong et al. combined DNA methylation, RNA-seq, and variant data from 116 TCGA tumors to predict recurrence within molecular subtypes, using *t*-tests to select differentially expressed genes and methylated regions and decision trees and random forests as classifiers [19]. On histology, AlZobi et al. used particle swarm optimization to tune the soft-voting weights of an ensemble of convolutional and transformer networks on tiles from 95 TCGA-UCEC whole-slide images [20]; the optimizer tunes ensemble weights and performs no feature selection, and the classes are histopathological categories, not the TCGA molecular subtypes. Closest to molecular subtyping from multi-omics data, the EMMA-STRAT framework integrates mRNA, miRNA, and DNA-methylation data from 433 TCGA patients to predict the TCGA genomic subtype and MSI status, benchmarks six classifiers, and validates on two external cohorts of the Clinical Proteomic Tumor Analysis Consortium (CPTAC) [21]; its feature selection relies on classical statistical and embedded methods (analysis of variance, and random-forest, support-vector-machine, and lasso rankings combined with recursive feature elimination) and does not involve a metaheuristic wrapper. Beyond endometrial cancer, hybrid ensemble feature selection on multi-omics and digital-pathology data has been used for prognostic biomarker discovery in pancreatic cancer, combining embedded selectors with wrapper-based recursive feature elimination [22].

Two pan-cancer studies frame the subtyping problem more broadly. Ellrott et al. applied five machine-learning approaches to multi-omic data from 8791 TCGA tumors spanning 106 subtypes in 26 cohorts, deliberately building models on small feature sets so that new samples from other studies could be assigned to existing TCGA subtypes [23]. Abbasi et al. compared 35 classifiers across 153 data sets, eight omics modalities, and 20 cancers; they attributed the limited comparability of earlier studies to non-standardized data-set configurations, diverse omics modalities, and inconsistent evaluation measures [24]. A review of multi-omics study design on TCGA listed class balance, feature selection, and the number of classes among nine factors that determine whether an analysis is robust [25], and a review of feature selection for tumor-subtype classification stressed the interpretability gains of compact panels [26]. Among the studies reviewed here, metaheuristic wrapper selection has not been evaluated for molecular subtyping of endometrial carcinoma from multi-omics data. What these studies share is a statistical prefiltering step that reduces the raw feature space to a candidate pool of manageable size before any model-based selection is attempted.

### 2.3. Metaheuristic Wrapper Feature Selection in Cancer Genomics

Wrapper feature selection scores a candidate subset by the cross-validated performance of a classifier trained on it [6]. Because the number of subsets grows exponentially with the number of features, the search is almost always delegated to a metaheuristic. Population-based optimizers are attractive here because they need no gradient, handle the discontinuous, noisy objective that cross-validation produces, and explore many subsets in parallel. A review of 91 cancer-classification studies reported particle swarm optimization, genetic algorithms, and ant colony optimization as the most common metaheuristics for feature selection and network optimization [7].

Several of the optimizers investigated here have published wrapper applications to high-dimensional cancer omics data whose structure is comparable to a multi-omics cohort; four of them are discussed in detail here. The Harris hawks optimizer [27] was combined with minimum-redundancy–maximum-relevance filtering to select genes for brain-cancer diagnosis from a 54,676-probe expression data set [8]; its fitness function is the weighted sum of the classification error and the fraction of selected genes. The binary gray wolf optimizer [28] was applied to a multi-omics breast-cancer data set after a neural-network prefilter [11]; together with a particle swarm optimizer that was applied to select features from multi-omics cancer data [12], it is, to our knowledge, one of the closest published precedents of a metaheuristic wrapper on multi-omics cancer data. Cuckoo search [29] was used for gene selection in microarray cancer classification [9], and the ant colony algorithm was applied to high-dimensional expression data with deliberately minimal prefiltering, its authors arguing that the algorithm does not require a small preselected gene set [10]; a continuous-domain formulation of the ant colony algorithm also exists [30]. Collectively, these studies show that the intersection of metaheuristic search and cancer omics is an active field, but also that comparisons across papers are confounded by differing prefilters, objectives, classifiers, and validation schemes. Comparative studies within a single protocol exist but are narrow: Hameed et al. compared binary particle swarm optimization, a genetic algorithm, and cuckoo search after a correlation filter on 12 cancer data sets [31]; Gürkan Kuntalp et al. compared six metaheuristic selectors with the Matthews correlation coefficient as the fitness function on imbalanced binary and three-class respiratory-disease data [32]; and Dhamercherla et al. compared their newly proposed eagle prey optimizer with 12 established optimizers on binary and multi-class microarray data [33]. These comparisons either included no more than six optimizers or accompanied the proposal of a new algorithm, and none of them was performed on multi-omics data. Among the studies reviewed here, none compares a large number of optimizers under one fixed protocol on a multi-class, imbalanced, multi-omics problem.

### 2.4. Objective Functions in Single-Objective Wrapper Feature Selection

A single-objective wrapper reduces feature selection to the maximization of one scalar function of a candidate subset, and the design of that function determines what the search will find. Since Kohavi and John [6], the function has been built from two ingredients: a classification term, estimated by training and validating a classifier on the candidate subset, and a parsimony term that discourages large subsets. The survey of Xue et al. covers the evolutionary-computation literature on this design [34]. The dominant formulation in the metaheuristic literature is the weighted sum used for binary gray wolf optimization by Emary et al., αε(S)+(1−α)|S|/N, where ε(S) is the classification error of the subset *S*, |S|/N is the fraction of the *N* available features it retains, and α is fixed close to one, typically 0.99 [28]. The same expression, with minor changes of notation, is used by the whale-optimization wrappers of Mafarja and Mirjalili [35], by the Harris hawks gene selector of Manhrawy et al. [8], and by most of the studies reviewed in Section 2.3. Three design decisions are hidden in this expression.

The first is the choice of the classification term. Almost all wrappers use the error rate, or equivalently the accuracy, of a single classifier. This choice is neutral only when the classes are balanced. Barros et al. compared accuracy, *F*-measure, area under the receiver operating characteristic curve (AUC), recall, and the relative accuracy improvement as fitness functions of a hyper-heuristic evolutionary algorithm that designs decision-tree induction algorithms, and they found that the best fitness function differed between balanced and imbalanced data; on imbalanced data, the six best-ranked variants used the *F*-measure, recall, or AUC, and the accuracy-based variants ranked seventh to ninth [36]. In feature selection specifically, Du et al. replaced accuracy by the extended geometric mean of per-class recalls in a genetic-algorithm wrapper for multi-class imbalanced data [37]; Namous et al. replaced it by the AUC and by the geometric mean in evolutionary and swarm-based wrappers on six binary imbalanced data sets [38]; Zhang et al. defined a modified *F*-measure that accounts for the risk introduced by imputing missing values and used it as the objective of a clustering-guided particle swarm optimizer for high-dimensional imbalanced data, mainly binary [39]; Tye et al. combined *F*-measure, geometric mean, and AUC in a penalty–reward fitness for binary microarray data [40]; and Yang et al. proposed a borderline-sensitive fitness that uses local density information to favor features near class margins on microarray data [41]. Outside the metaheuristic literature, Liu et al. used the reduction of *F*-measure optimization to a sequence of cost-sensitive problems to build an embedded feature selector for binary, multi-class, and multi-label data [42]. Most of these alternatives are binary measures, in which the *F*-measure refers to the positive class alone, or measures based on recall only; the multi-class *F*-measure optimized by Liu et al. is micro-averaged. The macro-averaged $F_{1}$ score, which combines precision and recall for every class and weights the classes equally, is a natural multi-class counterpart that we adopted here.

The second decision is how the classification term is estimated. A single hold-out split is cheap but noisy; *k*-fold cross-validation inside the search reduces the variance at *k* times the cost. If the folds are drawn from the outer training fold only then cross-validation also keeps the search free of leakage [15]. Because the search evaluates thousands of subsets, the noise of this estimate sets a floor below which differences between subsets are invisible to the optimizer; a parsimony term whose contribution per feature is smaller than that floor exerts no selection pressure. The third decision is the weight and form of the parsimony term. The linear term |S|/N with 1−α=0.01 is a convention rather than a calibrated trade-off, and its effect depends on the scale of the classification term: with an error rate near 0.1, removing one feature out of a few thousand changes the objective by less than $10^{-5}$. Some studies have added further terms—for example, a filter-based relevance or redundancy score [34]—and others have abandoned the weighted sum for a multi-objective formulation that returns a Pareto front of subsets [13,43]; the latter return a set of subsets instead of a single one and are not directly comparable with single-objective wrappers.

### 2.5. Class Imbalance in Feature Selection and Evaluation

Learning from imbalanced data is a mature subfield with well understood failure modes: a classifier, or a selection procedure driven by one, can achieve high overall accuracy while ignoring the minority class [44,45]. The literature reviewed above almost invariably uses accuracy as the classification term of the wrapper objective and as the headline metric. This is defensible for balanced benchmark data sets but not for molecular subtyping, where the minority class is the one of greatest clinical interest. Macro-averaged $F_{1}$, which averages the per-class $F_{1}$ scores with equal weight, is a standard remedy at evaluation time; placing it inside the objective function of the search is a direct way to prevent the search itself from discarding features that discriminate the rare class. Imbalance-aware wrapper objectives have been studied on microarray and tabular benchmarks. Du et al. replaced accuracy by an extended G-mean, the geometric mean of per-class recalls, in a genetic-algorithm selector with a support-vector classifier and reported equal or smaller subsets and higher per-class accuracy on the minority classes of multi-class benchmark data [37]. Tye et al. combined fuzzy feature clustering with a penalty–reward fitness that jointly optimizes the *F*-measure, G-mean, and AUC on highly imbalanced binary microarray data [40]. Yang et al. introduced a borderline-sensitive fitness function that uses local density information to favor features near class margins, together with a dual-phase feedback mechanism for progressive feature pruning, and they reported macro-averaged $F_{1}$ gains above 15% over six competing methods on 14 microarray data sets with imbalance ratios between 1.4 and 20 [41]. These works confirm that the fitness metric changes which features are selected; none of them, however, used the macro-averaged $F_{1}$ score as the classification term of the search objective, operated on multi-omics data, targeted molecular subtypes, or compared search algorithms under a common imbalance-aware objective. Complementary safeguards were stratified cross-validation, which guaranteed that every fold contained minority-class patients, and per-class reporting of performance.

## 3. Materials and Methods

### 3.1. Cohort and Data

All molecular data were downloaded from the Genomic Data Commons (GDC) TCGA-UCEC hub of the UCSC Xena platform [46]: gene expression from the STAR pipeline as $\log_{2}\left(\mathrm{FPKM}+1\right)$ (FPKM, fragments per kilobase of transcript per million mapped reads), miRNA expression as $\log_{2}\left(\mathrm{RPM}+1\right)$ (RPM, reads per million), Illumina 450 K DNA-methylation beta values, gene-level copy number from ASCAT3, whole-exome somatic mutation calls, and the clinical table. Molecular-subtype labels were taken from the TCGA PanCancer Atlas 2018 endometrial cohort in cBioPortal [47], which assigns each tumor to one of the four classes of the original TCGA study [1]: *POLE*-ultramutated, MSI-hypermutated, copy-number-low, and copy-number-high. For every modality only the primary-tumor sample (TCGA sample type 01) of a patient was used. We required every patient to have all five omics layers, the clinical variables, and a subtype label; this strict intersection yielded 369 patients (38 *POLE*, 105 MSI, 104 copy-number-low, 122 copy-number-high), and no patient was lost to a missing primary-tumor sample in any modality.

Mutation calls were reduced to a binary gene-by-patient matrix that recorded whether a gene carried at least one non-silent variant (missense, nonsense, frameshift, in-frame insertion or deletion, splice-site or start/stop-altering); synonymous, intronic, and untranslated-region calls were ignored. The clinical block held five variables derived from the GDC clinical table: age at diagnosis, International Federation of Gynecology and Obstetrics (FIGO) stage coded as an ordinal 1–4, and a one-hot encoding of histology (endometrioid, serous, other); the other columns of that table are identifiers, dates, or administrative fields. Tumor grade is reported as “Not Reported” for the whole cohort in the GDC table and was not used. Before any analysis, omics features that contained missing values or were constant across the cohort were removed; this step used no labels. Table 1 lists the resulting feature counts. Three patients lacked an age value; it was imputed with the median of the training fold in which the patient fell, never with a cohort-wide value.

### 3.2. Overview of POISE-X

**Figure 1:**
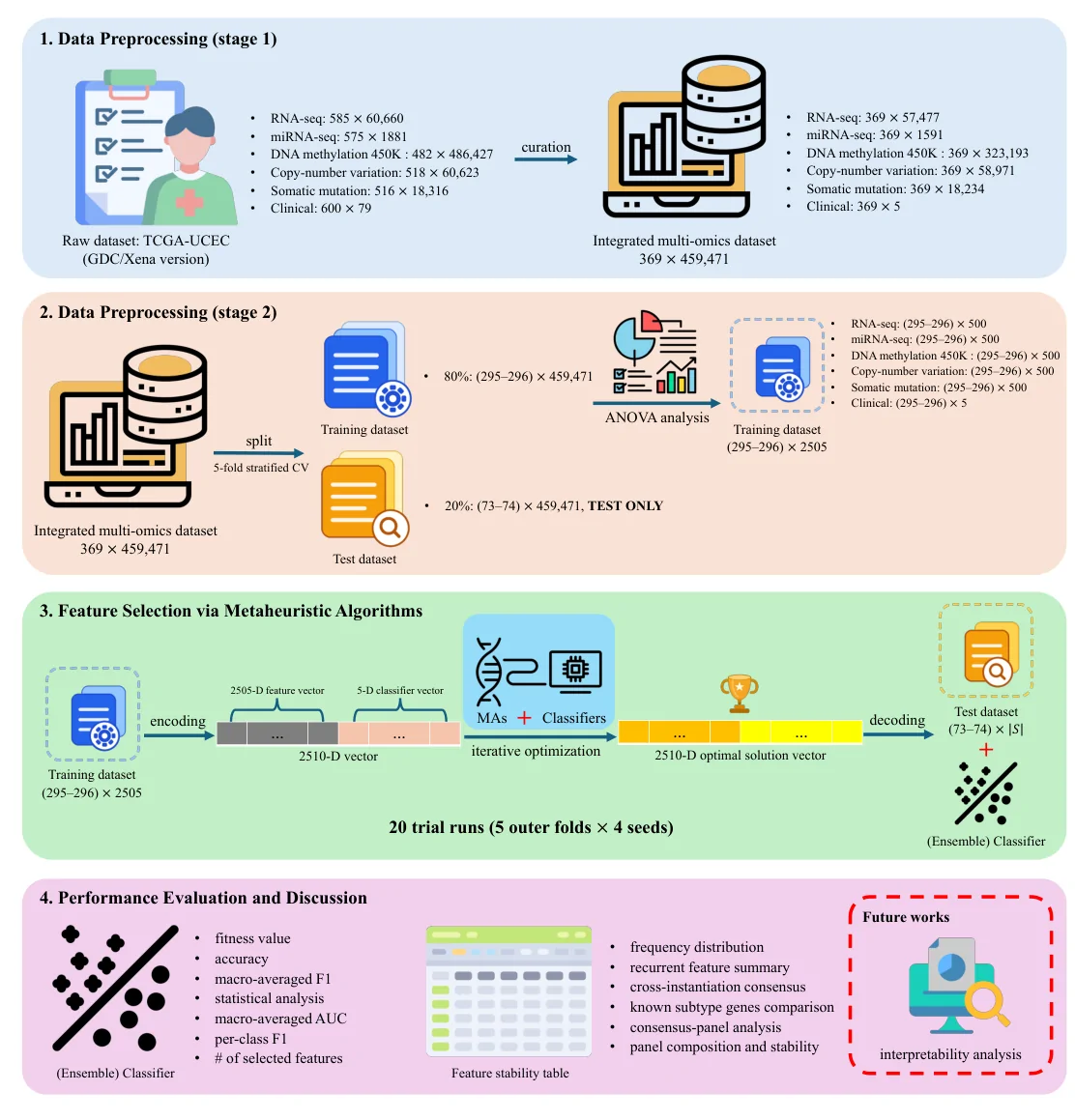
The POISE-X pipeline. Every data-dependent step (prefiltering, standardization, the wrapper search, and the classifier choice) was executed inside the outer training fold; the outer test fold was unseen until the final evaluation. The search engine X was exchangeable.

Figure 1 summarizes the pipeline. The cohort was split once into five stratified outer folds. Within each outer training fold, a label-dependent prefilter reduced the 459,471 features to a candidate pool of *N* features, a metaheuristic optimizer searched the combined space of feature subsets and classifier ensembles using a class-imbalance-aware fitness function evaluated by inner cross-validation, the best individual was decoded into a feature panel and a classifier set, and a final model was refitted on the whole outer training fold. Only then was the outer test fold used, once, to record the performance of that model. The optimizer, denoted X, was the only component that changed between instantiations; everything upstream and downstream of it was fixed.

We use “leakage-controlled” in the following sense: every data-dependent step—namely prefiltering, standardization, the wrapper search, and the choice of classifiers—was fitted within the outer training fold, and the outer test fold was used once, to score the resulting model. The choice among the 19 configurations, however, was made on the outer test folds, so the score of the chosen configuration was not an independent estimate; Section 4.7 evaluates this choice by leave-one-fold-out selection, and an unbiased estimate for a pre-specified configuration would require an external cohort.

### 3.3. Outer Cross-Validation and In-Fold Prefiltering

The outer split was a stratified five-fold partition (scikit-learn StratifiedKFold, shuffled, random seed 0) that was fixed once and shared by every instantiation and every run. Four outer training folds contained 295 patients and one contained 296; the corresponding test folds contained 74 patients and 73 patients, respectively, with 7 or 8 *POLE* tumors per test fold. Five folds was the largest number compatible with keeping a usable number of *POLE* cases in every test fold.

Within an outer training fold, the candidate pool was built as follows. In each omics block, features that were constant on the training rows were dropped. In the mutation block, only genes mutated in at least 3% of the training patients were retained, because rarer binary features could not be estimated by a wrapper on 295 or 296 patients. The remaining features of each omics block were ranked by the one-way analysis of variance (ANOVA) *F* statistic against the subtype label, and the top 500 per block were kept. The five clinical variables were always included. The pool therefore had N=5×500+5=2505 features. The prefilter was refitted in every outer fold from that fold’s training rows alone, so the pool differed between folds: of the 4257 features that entered at least one fold’s pool, 1156 entered all five, and two folds shared on average 1737 of their 2505 pooled features (69%).

### 3.4. Search Space, Decoding, and Classifier Pool

An individual was a vector $x\in {\left[0,1\right]}^{N+5}$. Coordinates 1,…,N corresponded to the pooled features and coordinates N+1,…,N+5 to five candidate classifiers. Decoding thresholded every coordinate at 0.5: the feature subset was $S=\{j\leq N:x_{j}>0.5\}$ and the classifier set was $C=\{c:x_{N+c}>0.5\}$. If *C* was empty then one classifier was drawn at random from the run’s random-number stream, anew at each evaluation. When |C|>1, the classifiers formed a soft-voting ensemble whose class probabilities were the unweighted mean of the members’ predicted probabilities; the predicted class was the arg-max. Thresholding a coordinate in [0,1] at 0.5 was a deterministic step transfer function, in contrast to the probabilistic S-shaped and V-shaped transfer functions of [48]; because it was applied identically to all optimizers, differences between instantiations arose from the search dynamics alone.

The five candidate classifiers were RF (100 trees), XGBoost [49] (50 trees, multi-class log-loss objective), an SVM with a radial-basis kernel and Platt-scaled probabilities, $L_{2}$-regularized multinomial LR (lbfgs solver, at most 1000 iterations), and KNN (k=5). All other hyperparameters were the scikit-learn 1.9 or XGBoost 3.2 defaults [49,50]. No classifier was tuned inside the search; the search chose among fixed classifiers.

### 3.5. Class-Imbalance-Aware Fitness Function

The fitness of an individual was

(1) $$F\left(x\right)=\alpha \;\left(1-\bar{F_{1}}\left(S,C\right)\right)+\left(1-\alpha \right)\;\frac{\left|S\right|}{N},\;\alpha =0.9,$$

to be minimized, where $\bar{F_{1}}\left(S,C\right)$ was the macro-averaged $F_{1}$ score of the ensemble *C* trained on the features *S*, estimated by stratified three-fold cross-validation inside the outer training fold. In each inner fold, a *z*-score standardization was fitted on the inner training part and applied to the inner validation part, the members of *C* were trained, and the macro-averaged $F_{1}$ of the soft-voted prediction was recorded; $\bar{F_{1}}$ was the mean over the three inner folds. The inner partition was drawn once per run from the run’s seed and kept fixed for all evaluations of that run, so that, for every individual whose classifier set *C* was non-empty, the fitness was a deterministic function of x within a run; for an individual with an empty *C*, the randomly drawn classifier, and hence the fitness, could differ between evaluations. An individual with |S|=0 received F=1. Macro-averaged $F_{1}$ weighted the four subtypes equally, so a subset that discarded the *POLE* class cost one quarter of the classification term regardless of the prevalence of that class. The objective followed the weighted-sum form of Section 2.4; the weight α=0.9 was the value used in [37,51] and gave the parsimony term ten times the weight it received under the common choice α=0.99. With α=0.9, the parsimony term contributed at most 0.1 to the objective, compared with a standard error of about 0.016–0.018 for the inner-fold estimate of macro-averaged $F_{1}$ at this sample size. This standard error was the standard deviation over the three inner folds divided by $\sqrt{3}$, computed for LR on the full candidate pool and on the ANOVA-ranked panel of 450 features and averaged over the outer folds; changing only the random inner partition moved the estimate by about 0.009. The search could therefore trade a substantial reduction in panel size for a small loss in $F_{1}$, whereas at α=0.99 the reward for removing half of the pool, 0.005, would fall below the noise of the inner estimate. In practice, panel size depended more on the optimizer than on α; the sensitivity of the results to α is reported in Section 4.8.

### 3.6. Candidate Optimizers and Search Protocol

Table 2 lists the 19 optimizers that instantiated X. All are the versions labeled “original” in MEALPY 3.0.3 [17], which followed the original publications except for minor implementation choices such as the inertia weight of PSO, run with the library’s default hyperparameters, on the box-constrained continuous problem ${\left[0,1\right]}^{N+5}$ with the objective of Equation (1). Four of them (HHO, GWO, CS, and ACO) are those whose applications to cancer omics data are discussed in Section 2.3, and PSO and DE have also been applied to cancer data [12,31,43]; the remaining 15 span the evolutionary, swarm-based, physics-based, and mathematics-based families. Every instantiation used a population of 50 and a budget of exactly 5000 fitness evaluations per search.

Each instantiation was run 20 times. Run i∈{0,…,19} used outer fold ⌊i/4⌋ and random seed 2026+98i; the seed controlled the optimizer, the inner partition, and the classifiers. The same 20 (fold, seed) pairs were used for every instantiation, so the results were paired across optimizers. After a search, the best individual was decoded; if its classifier set was empty then the classifier of the final model was drawn from a random-number generator re-initialized with the run’s seed and therefore did not need to coincide with the classifier drawn when that individual was scored during the search. For such an individual, the decoded solution was the panel *S* combined with a randomly drawn classifier, and the final classifier was a fresh draw under the same rule; the panel itself was unaffected. Because the search retained the individual whose draw scored best, the fresh draw was expected to lower rather than raise the outer-test score of an affected run. A deterministic rule, such as choosing the classifier with the largest classifier coordinate, would remove this mismatch; the rule was not changed after the experiment, so that all the reported runs shared one protocol, and the effect of the affected runs on the comparison was examined by excluding them (Section 4.4). A *z*-score standardization was then fitted on the whole outer training fold restricted to *S*, the classifiers in *C* were refitted on it, and the model was applied once to the outer test fold. We recorded the macro-averaged $F_{1}$, the macro-averaged one-versus-rest AUC computed from the soft-voted probabilities, the accuracy, the per-class $F_{1}$, the panel size |S|, the composition of *S* by block, the identity of *C*, the best fitness, and the convergence curve.

### 3.7. No-Selection Reference

To measure what the wrapper search contributed beyond the prefilter, each of the five classifiers, the soft-voting ensemble of SVM and LR, and the soft-voting ensemble of all five were trained on the full candidate pool of every outer training fold, with the same prefilter, standardization, and folds, and they were evaluated on the corresponding outer test fold. These seven reference models involved no search. As a filter comparator of the size of the compact wrapper panels, LR was also trained on the top 450 features of the in-fold ANOVA *F* ranking of the candidate pool (Section 3.9).

### 3.8. Statistical Analysis

The primary endpoint was the outer-test macro-averaged $F_{1}$. The 20 (fold, seed) blocks formed a paired design across the 19 instantiations. Because the four runs of a fold shared the same training and test patients, the 20 runs were not independent. The primary test of differences among the 19 instantiations was therefore the Friedman test on the 19×5 matrix of fold means [68]; the Friedman test on the 19×20 matrix of run scores is reported alongside it. Because the folds came from a single cohort and shared training data, they were not the independent data sets for which this test was recommended [68], and its *p*-values were therefore approximate. We then took the instantiation with the highest mean score as reference and compared it with each of the other 18 by a two-sided Wilcoxon signed-rank test on the 20 paired differences, correcting the 18 *p*-values with the Holm step-down procedure [69]. These pairwise tests are reported as an exploratory description only: they overstate the effective sample size, with five folds no pairwise test on fold means can reach p<0.0625, and they should not be read as a ranking of individual optimizers. We call an instantiation not significantly different from the reference when its Holm-adjusted p≥0.05; this denotes the absence of evidence for a difference, not evidence of equivalence.

To single out one compact configuration for detailed reporting, we applied the following rule: among the instantiations that were not significantly different from the reference, we chose the one whose searches reached the lowest mean value of the objective of Equation (1). The first step used the outer test folds and admitted only instantiations whose outer-test scores were not significantly below those of the best; the second step used only training-fold information, the objective that the search itself minimized, which combined the inner-fold macro-averaged $F_{1}$ with the panel size in the proportion fixed by α. Because the first step used the same outer test folds on which the instantiations were compared, the score of the selected configuration was subject to selection bias; Section 5 returns to this point, and all 19 rows are reported. To evaluate the rule itself, we also applied it in a leave-one-fold-out manner: for each outer fold *k*, the complete rule, from the choice of the reference through the Holm-corrected pairwise tests to the lowest mean search objective, was applied to the 16 runs of the other four folds, and the outer-test score on fold *k* of the configuration so chosen, the mean of its four runs on that fold, was recorded. The five resulting scores estimated the performance of the selection procedure itself, not that of a configuration chosen after seeing all folds. The runs of the other folds were trained on data that included the rows of fold *k*, but none of the scores used for the choice was computed on those rows.

Panel stability was quantified by the mean Jaccard index between the feature panels of all 190 pairs of runs of an instantiation, and by the selection frequency of every feature, i.e., the number of runs in which it was selected, together with the number of runs in which it entered the fold-specific candidate pool. As a chance reference for the Jaccard index, we computed for every pair of runs the expected Jaccard index of two random panels of the same sizes drawn from the two runs’ fold-specific pools, and we averaged it over the 190 pairs.

### 3.9. Consensus Panels and the Distribution of the Subtype Signal

Selection frequencies described which features recurred across runs, but they did not show whether the subtype signal was concentrated in those recurrent features or spread across the pool. We therefore tested fixed panels of the most recurrent features in a second experiment in which no ranking used the test rows of the fold on which it was evaluated: if the signal was concentrated, a small consensus panel should have approached the score of the full pool; if it was diffuse, consensus panels should have performed like other panels of the same size. For each outer fold *k*, only the searches of fold *k* itself were used, because they were the only searches whose training data excluded the test rows of fold *k*; the searches of the other four folds were trained on data that contained those rows, and using their panels to rank features for fold *k* would have leaked test information into the panel. For an instantiation, the four runs of fold *k* (one per seed) yielded four panels, and for the all-instantiation consensus the 76 runs of fold *k* yielded 76 panels. Every feature of the fold-*k* pool was scored by the number of those panels that contained it; the pool was ranked by this count, and the top *m* features were kept for m∈{10,20,50,100,200,300,450,600,1000,1500,2505}. LR, the classifier the search chose most often, was trained on the training rows of fold *k* restricted to these *m* features, with a *z*-score fitted on the training rows, and scored on the test rows of fold *k*. Two comparators of the same size *m* were evaluated in the same way: the top *m* of the ANOVA *F* ranking computed on the training rows, and random panels of *m* pooled features, averaged over 20 draws. The result was the outer-test macro-averaged $F_{1}$ as a function of *m*, averaged over the five folds.

### 3.10. Sensitivity Experiment Design

Four further analyses tested the robustness of the main results; all used the same outer folds, in-fold prefilter, and in-fold standardization as the main experiment: (i) Data layers: LR was trained on the 500 in-fold prefiltered features of each omics block alone, on the full candidate pool with one block left out at a time, and on the mutation block combined with one other omics block and the clinical variables. (ii) Surrogate of the clinical molecular classification: from the same TCGA data, three indicators that mirrored the routine assays were derived, namely a pathogenic *POLE* exonuclease-domain mutation, mismatch-repair deficiency—defined as a non-silent mutation of *MLH1*, *MSH2*, *MSH6*, or *PMS2* or as *MLH1* promoter hypermethylation—and *TP53* mutation. They were combined either by LR trained in each outer training fold or by the sequential rule of the current molecular classification [4], adapted from ProMisE [2], which checked *POLE* first, then mismatch-repair deficiency, then *TP53*, and assigned all remaining tumors to the copy-number-low group. The *POLE* indicator was the presence of 1 of the 11 pathogenic exonuclease-domain variants [70], *MLH1* promoter hypermethylation was a mean beta value above 0.3 over the 27 probes of the 450 K array within 500 bp of the *MLH1* transcription start site, and thresholds of 0.2 and 0.4 are reported as a sensitivity check; details are given in Section S3 of the Supplementary Materials. None of the three indicators used the subtype labels. (iii) Omics-only analysis: the five clinical variables were removed from the candidate pool (N=2500), and the no-selection reference with LR, the ANOVA-ranked panel of 450 features, and the searches of POISE-GWO and POISE-ACO were repeated with 20 runs each and the same seeds; LR trained on the five clinical variables alone showed how much subtype information they carry on their own. (iv) Parsimony weight: the searches of POISE-GWO and POISE-ACO were run with α∈{0.8,0.9,0.95,0.99}, with 20 runs each and the same folds and seeds; α=0.9 was the main experiment.

### 3.11. Implementation and Computational Resources

The pipeline was implemented in Python 3.11 with NumPy 1.26, SciPy 1.16, scikit-learn 1.9.0, XGBoost 3.2.0, and MEALPY 3.0.3. The 380 searches (19 instantiations × 20 runs) were executed on nodes with Intel Xeon Gold 6548Y+ processors, eight searches concurrently per 16-core allocation with two threads each. Divided by its 5000 evaluations, the search time of a single run ranged from 0.057 s to 2.936 s per evaluation, depending on |S| and on the classifiers in *C*, XGBoost and the SVM being the most expensive members; averaged over the 20 runs of an instantiation, it ranged from 0.069 s for POISE-FOX to 1.674 s for POISE-CS. The searches consumed 280 h of search time in total, each search using two threads. The 160 additional searches of the sensitivity analyses (Section 3.10) consumed a further 141 h of search time; one of them, a repeat of a search of the main experiment, reproduced its panel and scores exactly. LR fits can differ in the third decimal with the number of linear-algebra threads, so all reference models were fitted with the library defaults, as in the main experiment. The source code is available as described in the Data Availability Statement.

## 4. Results

### 4.1. Performance of the 19 Instantiations

Table 3 reports, for every instantiation, the outer-test macro-averaged $F_{1}$, macro-averaged AUC, accuracy, and panel size over the 20 runs, the Holm-adjusted *p*-value of the paired comparison with the reference instantiation, and the mean search time. On fold means, the primary analysis, the Friedman test rejected the hypothesis that the nineteen instantiations performed equally ($\chi_{18}^{2}=60.076$, $p=1.989\times 10^{-6}$); on the 20 runs, the corresponding values were $\chi_{18}^{2}=99.740$ and $p=2.472\times 10^{-13}$. POISE-ACO had the highest mean macro-averaged $F_{1}$ (0.900) and was the reference for the exploratory pairwise tests. In these run-level tests, four instantiations were not significantly different from it after Holm correction: POISE-DE (0.888), POISE-PSO (0.886), POISE-AO (0.882), and POISE-GWO (0.880), all with adjusted p=0.097. POISE-CS (0.883) and the remaining 13 had adjusted p=0.024 and p≤0.007, respectively. With five folds, however, a paired Wilcoxon test cannot reach p<0.0625, so the pairwise results above rested on treating the four seeds of a fold as separate blocks; they describe the data but should not be read as a ranking of individual optimizers, and an adjusted p≥0.05 is not evidence of equivalence.

**Figure 2:**
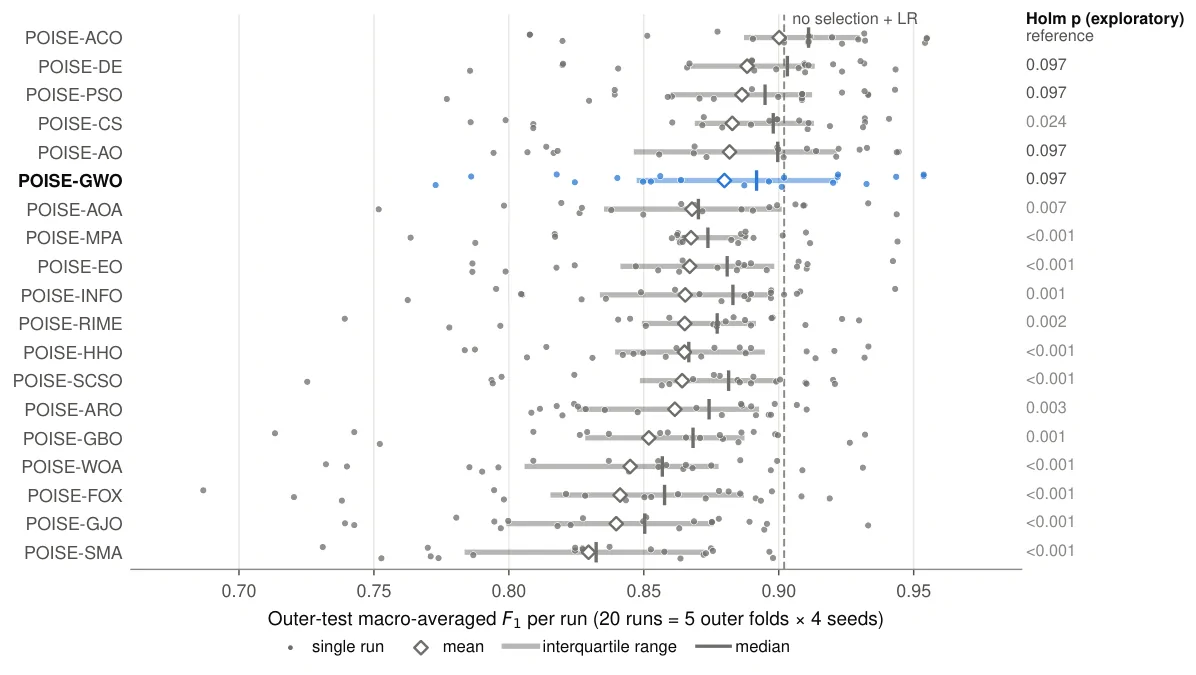
Outer-test macro-averaged $F_{1}$ of every run. Each row shows the 20 runs of one instantiation (points), their interquartile range (bar), median (tick), and mean (diamond); the dashed line is the no-selection reference with LR. The right-hand column gives the Holm-adjusted *p*-value of the paired Wilcoxon test against POISE-ACO, an exploratory test because the 20 runs were not independent (Section 3.8). POISE-GWO, the compact configuration singled out by the selection rule (Section 3.8), is labeled in bold and drawn in blue; all other instantiations are drawn in gray.

Figure 2 shows the 20 individual scores behind each row. The spread within an instantiation was large relative to the differences between instantiations: standard deviations of 0.036–0.065 corresponded to differences of two-to-four *POLE* tumors among the 73 or 74 patients of a test fold. The outer folds differed in difficulty for every instantiation; for POISE-GWO, for example, the fold means ranged from 0.804 (fold 3) to 0.931 (fold 4).

### 4.2. Performance Against Panel Size

**Figure 3:**
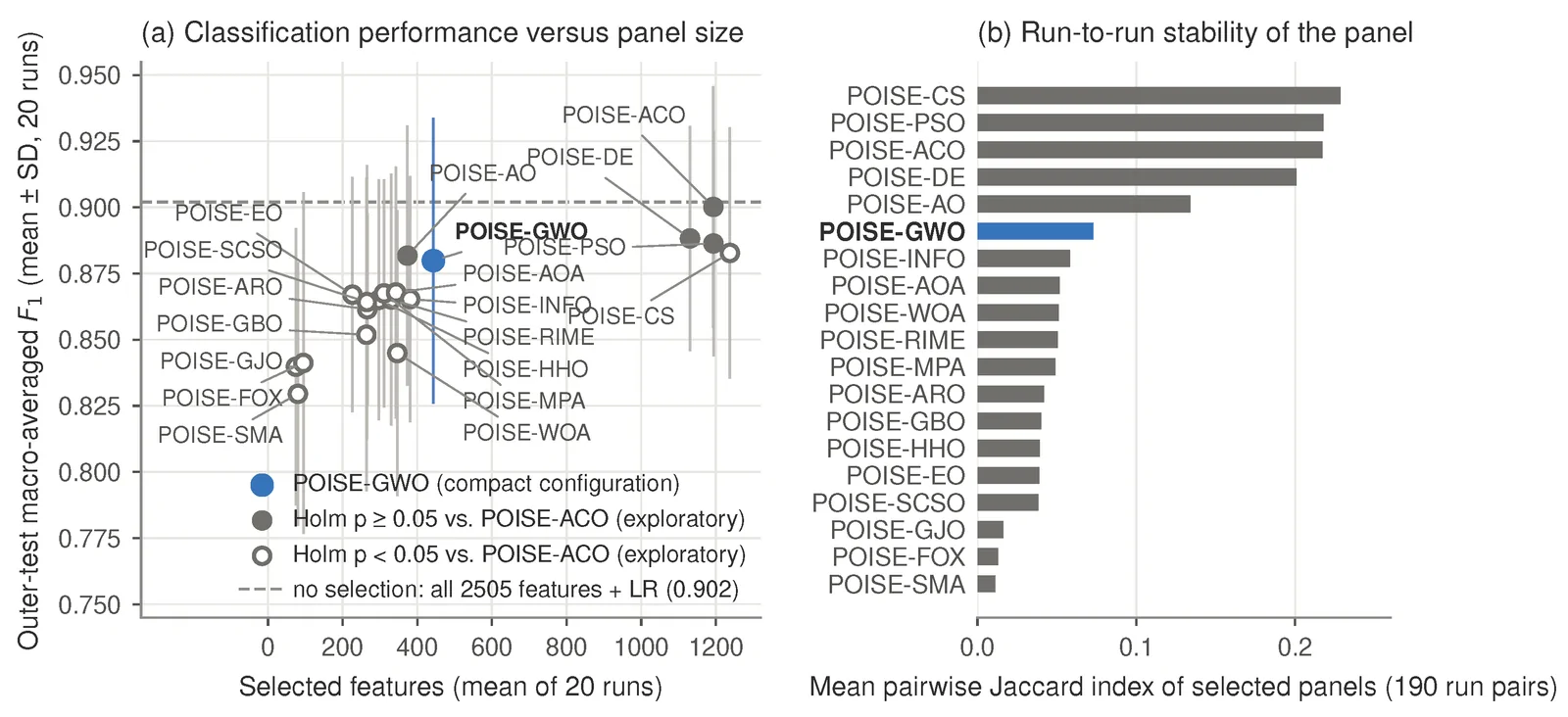
(**a**) Mean outer-test macro-averaged $F_{1}$ (±SD) against mean panel size for the 19 instantiations. Filled markers: Holm-adjusted p≥0.05 against POISE-ACO; hollow markers: p<0.05 (exploratory run-level tests; Section 3.8). The dashed line is the no-selection reference. (**b**) Mean pairwise Jaccard index between the feature panels of the 20 runs of each instantiation. In both panels, POISE-GWO, the compact configuration singled out by the selection rule (Section 3.8), is labeled in bold and drawn in blue.

The instantiations fall into two groups by panel size (Figure 3a). POISE-ACO, POISE-DE, POISE-PSO, and POISE-CS retained 1131–1238 features, between 45% and 49% of the pool, with small variation between runs. The other 15 retained 75–443 features. Across the instantiations, the mean panel size and the mean macro-averaged $F_{1}$ were strongly associated (Spearman ρ=0.851 over the 19 means), and no instantiation exceeded the no-selection reference of 0.902 obtained by LR on all 2505 pooled features; LR on the top 450 features of the in-fold ANOVA ranking scored 0.886, slightly above the POISE-GWO panels of similar size (Table 3). The four large-panel instantiations came within 0.002–0.019 of the reference with about half of the features; the compact instantiations lost 0.020–0.072 of macro-averaged $F_{1}$ relative to the reference for panels that were 2.551 to 16.545 times smaller than those of the large-panel instantiations.

Within the compact group, POISE-AO (374 features, 0.882) and POISE-GWO (443 features, 0.880) were the only instantiations that were not significantly different from POISE-ACO. Among the five instantiations not significantly different from it, the mean best objective reached by the searches was 0.053 for POISE-GWO, 0.085 for POISE-AO, 0.094 for POISE-ACO, 0.095 for POISE-DE, and 0.098 for POISE-PSO, so the selection rule of Section 3.8 designated POISE-GWO. It retained 18% of the candidate pool and 37% of the features retained by POISE-ACO while its macro-averaged $F_{1}$ was 0.020 lower, a difference that did not reach significance on 20 paired runs, although the fold mean of POISE-GWO was below that of POISE-ACO in all five folds, by 0.003–0.057. POISE-GWO was therefore one reasonable compromise between score and panel size, not the empirically best instantiation, and the absence of a significant difference on 20 non-independent runs is not evidence that the two were equivalent. Its panel size was consistent across runs, from 353 to 571 features with an SD of 52, and its search was the fastest of the five instantiations not significantly different from the best, with 0.451 h per run against 1.300 h for POISE-ACO and 2.200 h for POISE-AO. POISE-AO, the only other compact instantiation in this group, reached a higher objective with a panel whose size varied widely between runs, from 172 to 761 features with an SD of 161.

### 4.3. Search Behavior

**Figure 4:**
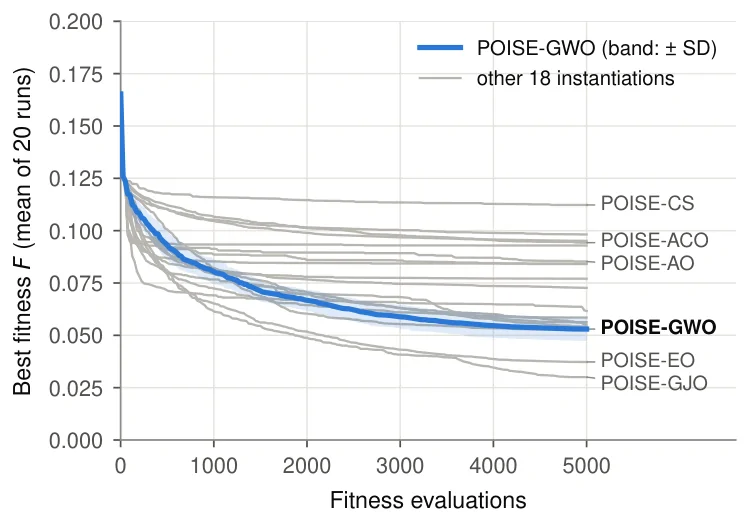
Mean best-so-far fitness *F* (Equation (1)) against the number of fitness evaluations for the 19 instantiations. POISE-GWO is highlighted with its ± SD band over 20 runs; the other 18 are drawn in gray, with a few labeled for orientation.

Figure 4 shows the mean best fitness against the number of evaluations. All the instantiations made most of their progress within the first 500 evaluations, after which the curves flattened. The large-panel instantiations plateaued at a fitness of 0.094–0.112, and most compact ones reached 0.030–0.085, the difference being largely the parsimony term 0.1|S|/N: with |S|/N≈0.475, that term alone contributed 0.047. POISE-GWO descended to 0.093 by evaluation 500 and 0.080 by evaluation 1000 and continued to improve until the end of the budget, reaching 0.053 with a narrow band between runs; POISE-AO, by contrast, stalled at 0.092 after 500 evaluations and ended at 0.085. That the instantiations with the lowest fitness values, POISE-GJO (0.030) and POISE-EO (0.037), did not reach the highest test scores shows that the inner-fold estimate of macro-averaged $F_{1}$ and the outer-test score are not interchangeable at this sample size.

### 4.4. Classifier Choice and Per-Class Performance

**Figure 5:**
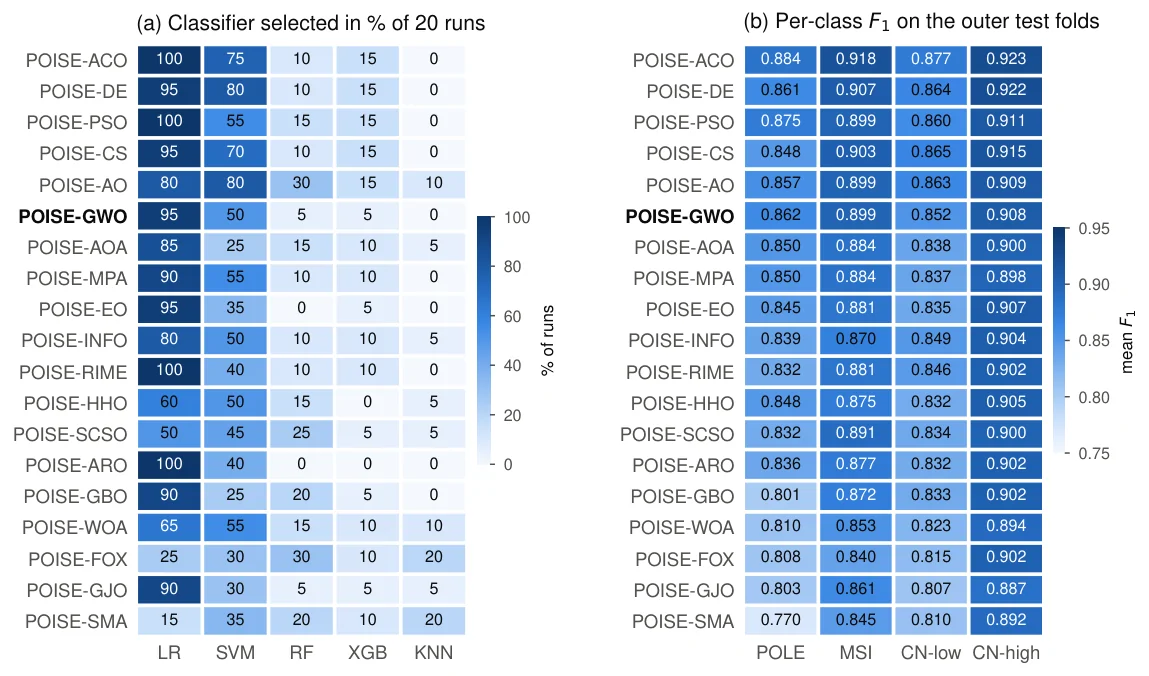
(**a**) Percentage of the 20 runs in which each candidate classifier was part of the final model. (**b**) Mean per-class $F_{1}$ on the outer test folds. LR, logistic regression; RF, random forest; XGB, XGBoost; KNN, *k*-nearest neighbors; CN, copy number.

The search chose LR in 80–100% of runs for most instantiations and the SVM in 25–80% (Figure 5a). RF, XGBoost, and KNN were rarely chosen, and the mean ensemble size was between 1.000 and 2.150 members. In 66 of the 380 runs, the best individual selected no classifier, and the final model used a randomly drawn one (Section 3.6); this happened in 14 runs of POISE-SMA, 13 of POISE-FOX, 9 of POISE-SCSO, 7 each of POISE-HHO and POISE-WOA, 4 of POISE-AO, 3 each of POISE-AOA and POISE-INFO, 2 each of POISE-GBO and POISE-GJO, and 1 each of POISE-MPA and POISE-CS, and never for POISE-ACO, POISE-DE, POISE-PSO, or POISE-GWO. For POISE-SMA and POISE-FOX, the classifier frequencies in Figure 5a therefore mostly reflect random draws. The final classifier of the affected runs was RF in 21, the SVM in 17, KNN in 16, XGBoost in 7, and LR in 5 runs, and their mean macro-averaged $F_{1}$ was 0.843, against 0.864 for the unaffected runs of the same 12 instantiations, in line with the expected downward effect (Section 3.6). Excluding the 66 affected runs, without any new search or model fit, did not change the main results. The mean macro-averaged $F_{1}$ of an affected instantiation changed by at most 0.015 (POISE-HHO, from 0.865 to 0.880); the highest mean among the affected instantiations, 0.891 for POISE-AO, remained below POISE-ACO (0.900) and the no-selection reference (0.902); and the range across the 19 instantiations remained 0.830–0.900. Because every run of POISE-SMA in fold 0 and of POISE-FOX in fold 4 was affected, the Friedman test on fold means could be repeated only for the other 17 instantiations; it remained significant ($\chi_{16}^{2}=46.416$, $p=8.395\times 10^{-5}$, against $\chi_{16}^{2}=50.510$, $p=1.901\times 10^{-5}$ for the same 17 with all runs). In the exploratory pairwise tests on the remaining pairs, POISE-CS (adjusted p=0.059) and POISE-SMA and POISE-FOX, with only six and seven remaining pairs (adjusted p=0.156), were no longer significantly different from POISE-ACO; POISE-GWO still had by far the lowest mean training objective in this larger group (0.053, against 0.083 or more for the others), so the selection rule of Section 3.8 would still single it out. The scores and ranks of the most affected instantiations, above all POISE-SMA, POISE-FOX, and POISE-SCSO, should nevertheless be read as properties of the optimizer together with this decoding rule. POISE-GWO chose LR in 95% of runs and the SVM in 50%; its final model was LR alone in 10 of 20 runs and the two-member ensemble of SVM and LR in 9. This preference agrees with the no-selection reference (Table 4), in which LR is the best single classifier on the full pool and the SVM-plus-LR ensemble is second; the search recovered the ranking of the classifiers without being told it.

Per-class $F_{1}$ (Figure 5b) was highest for the copy-number-high subtype, between 0.887 and 0.923 for every instantiation, and lowest for *POLE*, between 0.770 and 0.884, and copy-number-low, between 0.807 and 0.877. For POISE-GWO, the per-class values were 0.862 for *POLE*, 0.899 for MSI, 0.852 for copy-number-low, and 0.908 for copy-number-high; the standard deviation of the *POLE* score across runs, 0.129, was more than twice that of the other classes, as expected for a class with seven or eight test cases per fold.

### 4.5. Composition and Stability of the Selected Panels

**Figure 6:**
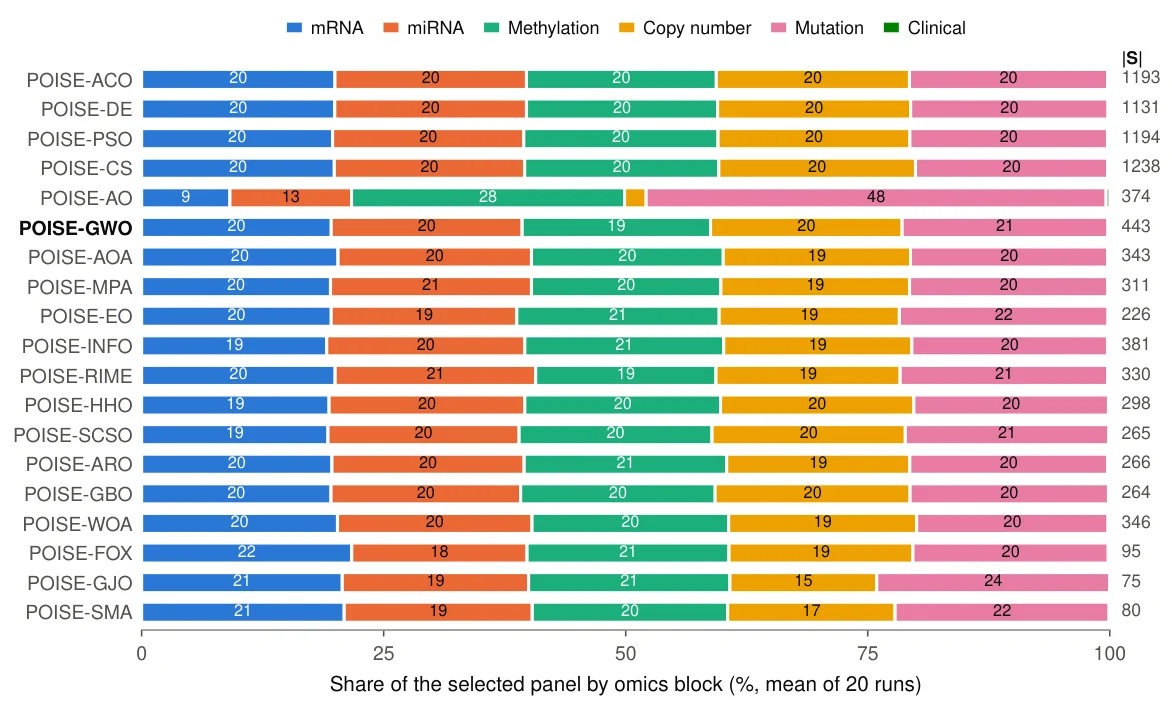
Share of each omics block in the selected panel, averaged over the 20 runs of each instantiation; the mean panel size |S| is given on the right. The candidate pool contained 500 features per omics block and five clinical variables.

Eighteen of the 19 instantiations, POISE-GWO among them, selected features from the five omics blocks in proportions close to those of the candidate pool, 15–24% per block against 20% in the pool (Figure 6); a POISE-GWO panel contained on average 87 mRNA, 87 miRNA, 86 methylation, 88 copy-number, and 94 mutation features. POISE-AO was the exception: its panels consisted on average of 48% mutation features and 28% methylation probes, with 9% mRNA, 13% miRNA, and 2% copy-number features. This composition was consistent across its runs and is discussed in Section 5. Clinical variables, five of the 2505 pooled features, never dominated a panel: the panels of an instantiation contained on average between 0.000 (POISE-GJO) and 2.700 (POISE-PSO) of them.

**Figure 7:**
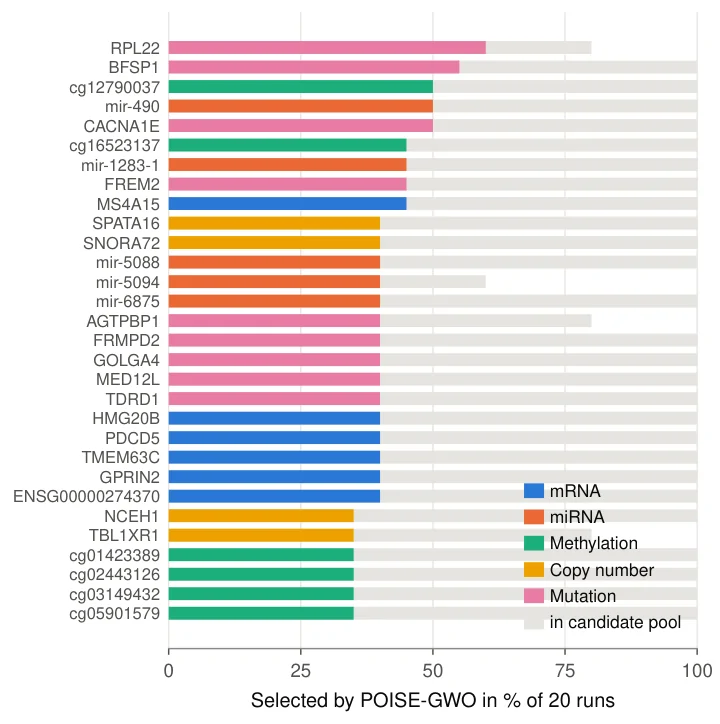
The 30 features most frequently selected by POISE-GWO, colored by data block. The light gray bar behind each feature shows the share of runs in which the feature was in the candidate pool. Recurrence does not establish biological or clinical relevance; see the caption of Table 5.

The run-to-run stability of the panels was low for every instantiation (Figure 3b). The mean pairwise Jaccard index was 0.201–0.229 for the four large-panel instantiations and 0.011–0.073 for the compact instantiations other than POISE-AO, for which it was 0.134. These values are close to the chance reference of Section 3.8, the overlap expected for random panels of the same sizes drawn from the same fold-specific pools, which was 0.202–0.226 for the large-panel instantiations and 0.070 for POISE-GWO, whose observed index was 0.073. The observed index exceeded its chance reference by at most 0.006 for every instantiation except POISE-AO, whose index of 0.134 compared with a chance reference of 0.054. The selection frequencies reflected the same picture: of the 3389 distinct features that POISE-GWO selected at least once, five were selected in at least half of the runs and none in more than 12 of the 20. Table 5 and Figure 7 list the most frequently selected features of POISE-GWO; they were drawn from all five omics blocks. The most frequent, *RPL22*, selected in 12 of the 16 runs in which it entered the pool, was also the feature most often selected across all 19 instantiations, as were *CACNA1E*, *MED12L*, and miR-490; *TP53*, *POLE*, and *PTEN* were selected in 5, 4, and 3 runs. Recurrence alone says nothing about whether these features carry the subtype signal, which is the question of the next section. Nor does it establish that any of them is causally or mechanistically related to a subtype, independently associated with it, or clinically useful: Table 5 and Figure 7 describe the behavior of the searches on this data set and should not be taken as lists of candidate biomarkers.

### 4.6. Distribution of the Subtype Signal over the Candidate Features

**Figure 8:**
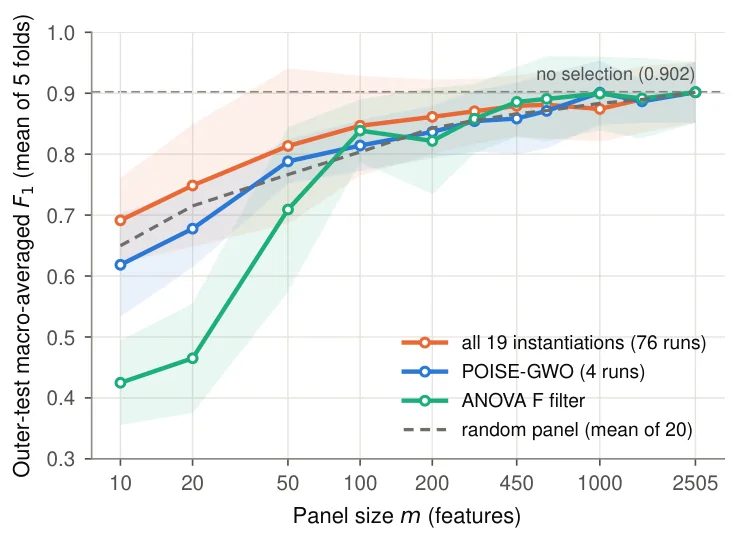
Outer-test macro-averaged $F_{1}$ of fixed panels of *m* features, mean over the five outer folds (bands: ±SD). Panels are ranked by the selection counts of each fold’s own searches, either over all 19 instantiations (76 panels per fold) or over the four POISE-GWO runs, and compared with the ANOVA *F* filter and with random panels of the same size. The dashed line is LR on all 2505 pooled features.

Figure 8 shows the outer-test macro-averaged $F_{1}$ of fixed panels of *m* features assembled from the selection counts of each fold’s own searches (Section 3.9). The consensus of all 19 instantiations, built from 76 panels per fold, was the best of the four panels compared at every m≤300: 0.691 at m=10, 0.813 at m=50, and 0.861 at m=200, against 0.650, 0.767, and 0.843 for random panels of the same size and 0.425, 0.709, and 0.822 for the ANOVA filter. Its advantage over random panels was 0.034–0.047 for m≤100 and shrank to 0.019 or less from m=200 onward. The consensus of the four POISE-GWO runs of a fold stayed close to the random panels at every size, at most 0.022 above them at m=50, where the two scored 0.788 and 0.767, and at most 0.037 below them at m=20, where they scored 0.678 and 0.715. Above m=450 every ranking converged toward the no-selection reference of 0.902, and the ANOVA filter, the weakest ranking at small *m*, was the strongest at m=450 and m=600, with 0.886 and 0.891. No panel in Figure 8 exceeded the reference at any size.

Two comparisons put these numbers in context. The fixed consensus panel of 450 POISE-GWO features scored 0.859, whereas the individual panels that POISE-GWO produced in single runs, of the same mean size, scored 0.880 (Table 3): the frequency ranking lost part of what the search found. And a panel of 450 features ranked by the ANOVA *F* statistic alone scored 0.886, slightly more than those single-run wrapper panels. Together, the flat curves of Figure 8, the closeness of consensus, filter, and random panels of equal size, and the ceiling set by the full pool indicate that in this cohort the subtype signal was not concentrated in a small set of features but spread over a large number of weakly informative and mutually redundant ones.

### 4.7. Evaluation of the Configuration-Selection Rule

To evaluate the configuration-selection rule itself, we applied it in a leave-one-fold-out manner (Section 3.8): for each outer fold, the rule was applied to the 16 runs of the other four folds, and the configuration it chose was scored on the held-out fold. POISE-ACO was the reference in every fold. The rule chose POISE-GWO in folds 0, 1, 2, and 4, where it reached outer-test macro-averaged $F_{1}$ of 0.929, 0.857, 0.877, and 0.931, and POISE-AO in fold 3, where it reached 0.809 and where POISE-GWO did not enter the group of instantiations not significantly different from the reference. The resulting estimate for the selection procedure was 0.881 ± 0.052 (Table S1), close to the 0.880 of POISE-GWO in Table 3 and below the 0.902 of the full-pool LR model. Choosing instead the instantiation with the highest mean score on the other four folds selected POISE-ACO in every fold and gave 0.900. The rule thus consistently favored the compact configuration; the optimism introduced by selecting it on the outer folds was small in this cohort; and favoring compactness cost about 0.019 in macro-averaged $F_{1}$ relative to the best large-panel configuration.

### 4.8. Results of Sensitivity Experiments

Data layers. No single omics block reached the score of the full candidate pool (Table S2). With LR, the mutation block alone reached a macro-averaged $F_{1}$ of 0.817, mRNA 0.801, methylation 0.730, miRNA 0.696, and gene-level copy number 0.348. Leaving out the mutation block lowered the score from 0.902 to 0.838, and the *POLE*$F_{1}$ from 0.897 to 0.716, whereas leaving out any other block lowered it by at most 0.028. The subtype information was therefore concentrated in, but not confined to, the mutation layer. Combined with the mutation block and the clinical variables, the methylation block reached 0.897 with 1005 features in total, within 0.005 of the full pool, whereas mRNA, miRNA, and copy number reached 0.880, 0.843, and 0.833.

Surrogate of the clinical molecular classification. Under the sequential rule, which has no parameters fitted to the subtype labels, the three indicators that mirror the routine assays assigned 338 of the 369 patients to their TCGA class, a proportion of 0.916 (Table S3). On the outer test folds, the rule reached a macro-averaged $F_{1}$ of 0.924 ± 0.030 and a *POLE*$F_{1}$ of 0.962, higher than the 0.902 of LR on the full multi-omics pool and than every wrapper panel on average. The difference from the full-pool model was concentrated in folds 2 and 3, where the surrogate scored 0.083 and 0.041 higher, whereas in the other three folds the two scores were within 0.012 of each other; LR trained on the three indicators obtained the same scores, with a macro-AUC of 0.959, lower than the 0.978 of the full-pool model. The advantage lies in the class assignments, not in the ranking of class probabilities, and it comes from the *POLE* and MSI classes: for the copy-number-high class, the surrogate reached an $F_{1}$ of 0.893, lower than the 0.915 of the full-pool model. Most disagreements, 17 patients, were copy-number-high tumors without a *TP53* mutation that the rule placed in the copy-number-low group. With *MLH1* methylation thresholds of 0.2 and 0.4 instead of 0.3, the rule reached 0.919 and 0.922.

Omics-only analysis. Because histology is strongly associated with the copy-number-high subtype, we repeated the main analyses without the five clinical variables (Table S4). The macro-averaged $F_{1}$ changed from 0.902 to 0.896 for LR on the full pool, from 0.880 to 0.875 for POISE-GWO with 430 features, and from 0.900 to 0.894 for POISE-ACO with 1177 features; in paired comparisons with the main experiment, neither search differed significantly, with p=0.812 and p=0.099. The ANOVA-ranked panel of 450 features was unchanged at 0.886, because no clinical variable ranked among its features in any fold. The $F_{1}$ of the copy-number-high class was 0.903 for POISE-GWO and 0.914 for POISE-ACO without the clinical variables, against 0.908 and 0.923 with them, and LR on the five clinical variables alone reached only 0.378. The reported performance therefore did not rest on histology.

Parsimony weight. Between α=0.8 and α=0.99, the panels of POISE-GWO grew from 346 to 479 features and those of POISE-ACO from 1123 to 1228 features (Table S5, Figure S1), so the gap between the compact and the large-panel search persisted at every value. The macro-averaged $F_{1}$ ranged from 0.869 to 0.881 for POISE-GWO and from 0.883 to 0.900 for POISE-ACO, and no configuration exceeded the no-selection reference of 0.902 at any value of α. POISE-ACO scored highest at α=0.9; at the other values it exceeded POISE-GWO by only 0.006–0.014.

## 5. Discussion

### 5.1. Comparison Experiments Analysis

The most informative result of this benchmark is a negative one: no metaheuristic wrapper improved on a linear model trained on the full candidate pool, and a univariate ANOVA filter truncated to the size of the best compact panels performed at least as well as those panels. On this cohort, the wrapper search therefore reduced the panel size without adding discriminative power, and a simple filter achieved a similar reduction.

Three results underlie this conclusion. First, the choice of optimizer matters: under an identical encoding, objective, classifier pool, budget, and evaluation protocol, the 19 instantiations spanned 0.071 in macro-averaged $F_{1}$ and a sixteen-fold range of panel size, and the Friedman test on fold means rejected equality with an approximate *p*-value of $1.989\times 10^{-6}$. An application paper that adopted one optimizer by convention would have reported a result somewhere in this range without knowing where. Second, the ranking by classification score is largely a ranking by panel size: the 4 instantiations that kept about half of the pool score highest and came within 0.019 of the no-selection reference; the 15 that compressed further lost between 0.020 and 0.072. Third, no instantiation improves on LR applied to the whole candidate pool. On this cohort, the wrapper search did not add discriminative power beyond the univariate prefilter; what it added was compression, from 2505 features to a few hundred, at a measurable but statistically non-significant cost for the best compact instantiations, although the top 450 features of the ANOVA ranking alone scored 0.886 with LR (Section 4.6).

The second and third results had a common cause. With 295 or 296 training patients and three inner folds, the standard error of the inner estimate of macro-averaged $F_{1}$ was about 0.016–0.018 (Section 3.5), whereas removing one feature changed the parsimony term of Equation (1) by $0.1/2505=3.992\times 10^{-5}$. The search therefore received a clear signal only from changes that moved many features at once, and it could not distinguish a subset that was truly better from one that happened to fit the inner folds. Optimizers whose update rules push coordinates toward the boundaries of the unit hypercube, such as GWO, EO, and HHO, produce sparse individuals early and then hold them; optimizers whose moves recombine or perturb around the population mean, such as DE, PSO, and ACO, keep coordinates near their random initialization at about 0.5 and decode into panels of half the pool. The panel size an instantiation reports is thus as much a property of its search dynamics as of the data, which is why we regard the comparison as a whole, more than the result of any single optimizer, as the informative output of the pipeline. The sensitivity analysis supports this reading: the gap in panel size between POISE-GWO and POISE-ACO persisted for every parsimony weight from 0.8 to 0.99 (Section 4.8).

### 5.2. The Compact Configuration POISE-GWO

POISE-GWO is reported as one performance–compression trade-off within this benchmark, not as the empirically best instantiation or as a validated model. We selected POISE-GWO because, among the five instantiations that are not significantly different from the best on the outer test folds, it is the one whose searches reach the lowest value of the objective they were asked to minimize. Three further properties characterize this configuration, none of which entered the rule. Its panel size is consistent across runs, so a user can expect a panel of 350–570 features from a single search. Its search is the fastest of these five instantiations, a third of the cost of POISE-ACO and a fifth of that of POISE-AO, because the gray wolf update pushes coordinates toward the bounds of the unit hypercube early and thereafter evaluates sparse individuals. Its panels are drawn from the five omics layers in proportion to their presence in the pool, so the compression it achieves is not the result of discarding a data type. Three of the five features it selects most often, *RPL22*, miR-490, and *CACNA1E*, are also the three features most often selected across all 19 instantiations; this recurrence describes how the searches behaved on this data set and says nothing about the biological relevance of these features. We do not, however, present any selected feature as a validated marker. The consensus experiment of Section 4.6 shows why: a fixed panel of the most frequently selected features was at most 0.047 better than a random draw of the same size and within 0.019 of it from 200 features onward, and it was a worse panel than the ones the search produced in a single run. Recurrence may have reflected individual informativeness within this data set, but it did not identify a compact set that carried the subtype signal on its own. We read this as a property of the data set: in the TCGA-UCEC cohort the subtype signal is spread over a large number of weakly informative and mutually redundant features, so that many different panels of a few hundred features are nearly interchangeable, the identity of any one panel is unstable, and no wrapper panel improves on a linear model that uses the whole candidate pool. The same property explains three other observations of this study: that LR on the entire pool was never beaten, that the wrapper reduced panel size without improving accuracy, and that the run-to-run Jaccard index of every instantiation was low. It also defines what POISE-X delivers on such data: a panel whose predictive value is estimated on held-out patients and whose size is a fraction of the pool, not a list of individually validated features. Multi-omics cohorts of other tumor types may have a more concentrated signal, and the consensus experiment of Section 3.9 is a direct way to find out.

A panel of several hundred molecular features measured on five platforms is not a diagnostic panel, and we do not propose it as one. POISE-GWO is reported as the configuration that, within this benchmark, offered the most favorable combination of score and panel size among the instantiations not significantly different from the best; its score was below that of the full-pool LR model, and a much simpler ANOVA filter reaches a comparable score at the same size.

The comparison also exposed a property of one optimizer that would have gone unnoticed in a single-optimizer study. POISE-AO, the other compact instantiation in this group, concentrates 48% of its panels on the mutation block and 28% on the methylation block and nearly ignores copy number at 2%, whereas every other instantiation samples the blocks close to evenly, at 15–24% per block. We attribute this to the search behavior of the Aquila optimizer rather than to the data. Its narrowed-exploration move perturbs a solution along a spiral whose angle and radius are functions of the coordinate index, so the perturbation applied to coordinate *j* depends on *j* [60]. Because the five omics blocks occupied contiguous ranges of coordinates in our encoding, the move acted differently on different blocks. The other 18 optimizers treated coordinates exchangeably. This was a case where an optimizer’s inductive bias, invisible on standard benchmark functions, becomes visible on a structured feature space, and it argues for reporting panel composition alongside panel size in any wrapper study.

### 5.3. Scope of the Study: Label Reconstruction Versus Clinical Utility

The task addressed in this study was technical: to measure how well, and with how many features, high-dimensional multi-omics data reconstruct the four molecular subtypes of the TCGA classification. The clinical relevance of these subtypes for risk stratification and adjuvant-treatment decisions has been established in other studies, and a model that reproduces them can at most inherit that relevance; it cannot add to it. We did not evaluate whether the selected panels carry information on survival, recurrence, or treatment benefit beyond the subtype labels, and the TCGA-UCEC cohort, with its limited follow-up and non-randomized treatment, is not suited to that question. The results should therefore not be read as evidence that the selected panels can guide treatment.

The four TCGA classes are themselves defined by genomic properties, namely *POLE* exonuclease-domain mutation, microsatellite instability and hypermutation, and the extent of somatic copy-number alteration [1], and gene-level mutation, methylation (including the *MLH1* promoter), and copy-number features are among the inputs of the pipeline. The scores reported here therefore measure how well high-dimensional molecular data reconstruct an existing classification; they are not evidence of a new, treatment-relevant molecular signature. This is appropriate for a benchmark of feature-compression strategies, but it bounds what the selected panels can mean clinically.

The five data layers used here are not available in routine care, and the benchmark was not designed to replace clinical molecular typing. In routine practice, the four molecular groups are assigned by *POLE* exonuclease-domain sequencing and immunohistochemistry for mismatch-repair proteins and p53, a combination that is inexpensive and widely validated [2,3]. A surrogate of this classification built from three indicators in the TCGA data reached a macro-averaged $F_{1}$ of 0.924, on average above every multi-omics model of this study (Section 4.8), and no single omics layer matched the combination of all five (Table S2). Within the scope of this study, the multi-omics panels therefore offer no practical advantage over established clinical assays; their value lies in the methodological comparison of feature-selection strategies on a high-dimensional, imbalanced problem.

### 5.4. Class Imbalance and the Objective

The *POLE* subtype, the rarest and clinically the most consequential, was the class with the most variable $F_{1}$ for every instantiation and the lowest mean $F_{1}$ for 11 of the 19, and the class on which the reference classifiers differed most (0.721–0.897 in Table 4). Placing macro-averaged $F_{1}$ in the objective meant that the search could not trade this class away for overall accuracy without a proportional loss of fitness: the accuracy and macro-averaged $F_{1}$ of every instantiation in Table 3 differed by less than 0.015, whereas under an accuracy objective, misclassifying every *POLE* tumor would cost only about ten points. Such a trade would be clinically indefensible regardless of the accuracy it bought: *POLE*-ultramutated status is precisely the distinction that changes adjuvant-treatment decisions, so a selection procedure that discards it in exchange for overall accuracy defeats the purpose of subtyping. We did not, however, run the same search with an accuracy-based objective, so the effect of this choice on the selected panels was not measured; LR trained on the full pool, with no imbalance-aware step, already reached a *POLE*$F_{1}$ of 0.897 (Table 4), higher than the mean *POLE*$F_{1}$ of any instantiation.

### 5.5. Limitations

The set of instantiations from which POISE-GWO was selected was defined on the same outer folds on which the instantiations were compared. Although the final choice within that set used only the training-side objective, the reported score of 0.880 was the score of a configuration chosen post hoc among 19 and may be optimistically biased; what the data support is that on 20 paired runs it could not be distinguished from the best instantiation. The leave-one-fold-out evaluation of the selection rule (Section 4.7) added a further level of validation around the selection step and suggests that the optimism introduced by selecting POISE-GWO on the outer folds is small in this cohort. This estimate was, however, internal and not fully nested, because the runs of the other folds were trained on data that included the held-out rows: POISE-GWO was a configuration selected within this benchmark, not a pre-specified and externally validated model. No external multi-omics endometrial cohort with matched modalities and TCGA subtype labels was available to us, so the pipeline has been validated only internally; an unbiased estimate for a pre-specified configuration, and confirmation of the performance of any configuration, would require an independent cohort.

The 20 runs per instantiation are not independent: the four runs of a fold shared their training and test patients, so the paired Wilcoxon tests, which treated them as separate blocks, overstated the effective sample size; on fold means alone, the Friedman test remained significant, with a *p*-value that was approximate because the folds shared training data, but no pairwise comparison could reach p<0.0625. Differences of about 0.020 in mean macro-averaged $F_{1}$ were not resolved, and the group of instantiations not significantly different from the best could shrink or grow with more folds or runs. In 66 of the 380 runs the best individual selected no classifier, and the classifier of the final model was then drawn independently of the one used to score that individual during the search (Section 3.6); among the five instantiations not significantly different from the best, only four runs of POISE-AO were affected, but the scores of the more strongly affected instantiations, above all POISE-SMA and POISE-FOX, may not have represented their searches. Excluding the affected runs left the main results unchanged (Section 4.4), but it removed non-random runs and could not show how these optimizers would have searched under a deterministic rule. The prefilter kept a fixed 500 features per block; we did not vary this number, and a different pool would change both the panels and possibly the ranking. The reading of the consensus experiment also depends on the feature representation: the TCGA classes are defined by genome-wide properties, namely mutation burden, microsatellite instability, and the extent of copy-number alteration [1], which the gene-level mutation and copy-number features used here carry only piecemeal. Aggregate features such as the tumor mutational burden, the fraction of the genome altered, *POLE* exonuclease-domain hotspot status, or *MLH1* promoter methylation were not included and might concentrate the signal in far fewer features, as the three-assay ProMisE classifier suggests [2]; the surrogate analysis of Section 4.8 supports this: three such indicators reached a macro-averaged $F_{1}$ of 0.924, higher on average than any panel selected from the 2505 candidate features. Two of the most frequently selected methylation probes, cg12790037, listed in Table 5, and cg14598950, tied for fourth-most-often-selected over all 19 instantiations, lie in the *MLH1* promoter and are among the probes from which the surrogate’s methylation indicator is computed. The run-to-run stability of the panels was low, with no feature selected in every run of any instantiation, and we deliberately did not impose a selection-frequency cut-off; a stable core panel, if one exists, will have to be defined by further runs or by aggregation across instantiations. Finally, the validation in this study applies to whole panels; individual features were not validated. The predictive value of each selected panel was measured on held-out patients, but the relevance of any individual feature to a subtype has not been established, and on a cohort whose signal is as diffuse as this one, recurrence across runs cannot establish it. Whether individual features merit biological follow-up is a separate question that would require external cohorts or functional evidence. The study demonstrates the predictive utility of panels within the TCGA-UCEC data set. It does not establish that individual selected features are causal, mechanistically relevant, clinically useful, or independently associated with a molecular subtype, and, given the low run-to-run stability and the small advantage of consensus panels over random panels, recurrence cannot establish this. Validation in an independent data set would be required before any of the recurrent features could be considered a potential biomarker. The sensitivity to the parsimony weight was assessed for two instantiations only, POISE-GWO and POISE-ACO, one from each panel-size group; the other 17 optimizers may respond differently to α.

### 5.6. Future Work

POISE-X re-uses existing optimizers unchanged; no optimizer was modified or proposed in this work. The comparison shows that the feature-selection problem it defines has properties that general-purpose optimizers handle unevenly: a search space of several thousand dimensions in which coordinates are decoded by a threshold, a noisy objective whose resolution is coarser than the parsimony reward for a single feature, and a block structure that at least one optimizer treats non-exchangeably. Given that no wrapper panel improved on the full-pool LR model and that a matched-size filter compressed about as well, a new optimizer is not the most pressing next step. More informative steps are the evaluation of the selected panels, the filter baselines, and the surrogate of the clinical classification in an independent multi-omics cohort, and the inclusion of aggregate features such as the tumor mutational burden, the fraction of the genome altered, *POLE* hotspot status, and *MLH1* promoter methylation, together with a comparison against filter baselines of the same size. An optimizer designed for these properties, for example one with an explicit sparsity operator and a noise-aware acceptance rule, could then be plugged into the same search-engine slot. The pipeline was built so that such an optimizer can be evaluated against the 19 reported here under identical conditions. Future versions should decode an empty classifier set deterministically, for example by taking the classifier with the largest classifier coordinate, so that the evaluated model always coincides with the scored solution.

A second line of future work concerns the features themselves. This study validated whole panels rather than individual features, and the recurrent features of Table 5 are reported as observations that should not be read as markers. Establishing whether any of them is individually relevant to a subtype will require analyses that this paper did not attempt: attribution of the final model’s predictions to individual features, for example with SHAP values, together with a check that the direction of each attribution agrees with the known biology of the subtype; cross-referencing of the recurrent features against curated cancer-gene resources and pathway enrichment against gene sets of known relevance to endometrial carcinoma, with the size of each panel controlled for; annotation of the recurrent methylation probes and miRNAs to their target genes; and, above all, evaluation of the selected panels on an independent multi-omics cohort. On a cohort whose subtype signal is as diffuse as this one, such analyses would also show whether the recurrent features are informative in their own right or merely the most stable representatives of larger redundant groups.

## 6. Conclusions

We presented POISE-X, a leakage-controlled benchmarking pipeline for multi-omics feature selection and reconstruction of the TCGA molecular subtypes of endometrial carcinoma, in which prefiltering, standardization, and feature selection are performed separately within each outer training fold and in which the metaheuristic search engine is an interchangeable component and the wrapper objective is the macro-averaged $F_{1}$ score with a parsimony penalty. We instantiated the search engine with 19 optimizers from the MEALPY library and evaluated all of them under one protocol on 369 TCGA-UCEC patients with 20 paired runs each. The instantiations differed significantly; their classification scores tracked their panel sizes; and none exceeded LR on the full candidate pool, so on this cohort the search reduced the number of features without improving accuracy, and a simple ANOVA filter matched the best compact wrapper panels at a comparable size. Applying the selection rule of Section 3.8, we singled out POISE-GWO, the gray-wolf-optimizer instantiation, as one performance–compression trade-off within this benchmark: it retained 443 of 2505 candidate features with a macro-averaged $F_{1}$ of 0.880 that was not significantly different from that of the best instantiation, reached the lowest search objective and the shortest search time of the five instantiations not significantly different from the best, and drew its panel evenly from the five omics layers; a leave-one-fold-out evaluation of the rule gave a macro-averaged $F_{1}$ of 0.881. A consensus-panel experiment showed that panels of the most recurrent features perform at most 0.047 better than random panels of the same size and within 0.019 of them from 200 features onward: in this cohort the subtype signal is spread over a large number of weakly informative and mutually redundant features, which is why no panel beats the full pool and why the value of the search lies in compression, although an ANOVA filter truncated to a similar size compresses as well. We reported all 19 rows because the selection was made on the same folds as the comparison. These conclusions held without the clinical variables and across parsimony weights in the two searches tested, and a surrogate built from three indicators that mirrored the routine clinical assays reached a higher macro-averaged $F_{1}$ than any multi-omics panel. The panels reconstructed an existing classification; they were neither diagnostic assays nor validated biomarkers, and external validation would be required before any selected feature could be considered a potential biomarker. The next steps are external validation in an independent multi-omics cohort and the use of aggregate features, compared with filter baselines of the same size; an optimizer designed for the specific structure of this problem can then be evaluated through the same slot against the results reported here.

## Figures and Tables

**Table 1:** Feature counts per data block after label-free cleaning. Methylation probes and copy-number genes with any missing value were removed, and constant features were removed in every block. For the clinical block, the five variables (age, FIGO stage, and a three-level histology encoding) were derived from 3 of the 79 columns of the GDC clinical table; the remaining columns are identifiers, dates, administrative fields, or, in the case of grade, not reported. For the mutation block, the raw count was the number of genes with at least one non-silent variant in the 369 patients; over all 516 sequenced samples, 18,316 genes carried such a variant.

Block	Raw Features	Removed	Retained
mRNA expression (RNA-seq)	60,660	3183	57,477
miRNA expression	1881	290	1591
DNA methylation (450K)	486,427	163,234	323,193
Copy number (gene level)	60,623	1652	58,971
Somatic mutation (non-silent, 0/1)	18,234	0	18,234
Clinical (derived) ^a^	79	76	5
Total			459,471

^a^ Unlike the omics rows, Removed and Retained do not sum to Raw for this row: of the 79 raw clinical columns, 76 are unused identifiers, dates, administrative fields, or the not-reported grade field (Removed), and only 3 (age, FIGO stage, histology) are kept; histology is then one-hot-encoded, so the 3 kept columns expand to 5 retained features.

**Table 2:** The 19 optimizers investigated as instantiations of the POISE-X search engine, in order of the year of their original publication. All were run in the versions labeled “original” in MEALPY [17], with the library’s default hyperparameters.

Abbreviation	Optimizer	Year	Reference
PSO	particle swarm optimization	1995	[52]
DE	differential evolution	1997	[53]
ACO	ant colony optimization for continuous domains	2008	[30]
CS	cuckoo search	2009	[29]
GWO	gray wolf optimizer	2014	[54]
WOA	whale optimization algorithm	2016	[55]
HHO	Harris hawks optimization	2019	[27]
EO	equilibrium optimizer	2020	[56]
MPA	marine predators algorithm	2020	[57]
SMA	slime mold algorithm	2020	[58]
GBO	gradient-based optimizer	2020	[59]
AO	Aquila optimizer	2021	[60]
AOA	arithmetic optimization algorithm	2021	[61]
ARO	artificial rabbits optimization	2022	[62]
GJO	golden jackal optimization	2022	[63]
INFO	weighted mean of vectors	2022	[64]
RIME	RIME optimizer	2023	[65]
SCSO	sand cat swarm optimization	2023	[66]
FOX	FOX optimizer	2023	[67]

**Table 3:** Outer-test performance of the 19 instantiations of POISE-X and of the no-selection reference (all 2505 pooled features with LR) and of LR on the top 450 features of the in-fold ANOVA ranking. Mean ± standard deviation (SD) over 20 runs (five outer folds × four seeds); the two reference models were over the five outer folds. |S| was the number of selected features out of 2505. Holm *p* (exploratory): two-sided paired Wilcoxon test against POISE-ACO, the instantiation with the highest mean macro-averaged $F_{1}$, Holm-corrected over 18 comparisons; the 20 runs were not independent (Section 3.8). Rows follow the order of Table 2; the best value in each column among the 19 instantiations is in bold.

Instantiation	Macro-Averaged $F_{1}$	Macro-AUC	Accuracy	|S|	Holm *p*	Time (h)
POISE-PSO	0.886 ± 0.043	**0.979 ± 0.012**	0.888 ± 0.035	1194 ± 32	0.097	1.606
POISE-DE	0.888 ± 0.043	0.978 ± 0.014	0.894 ± 0.034	1131 ± 37	0.097	0.922
POISE-ACO	**0.900 ± 0.046**	**0.979 ± 0.013**	**0.904 ± 0.034**	1193 ± 22	–	1.300
POISE-CS	0.883 ± 0.048	0.978 ± 0.014	0.890 ± 0.037	1238 ± 20	0.024	2.324
POISE-GWO	0.880 ± 0.054	0.975 ± 0.012	0.884 ± 0.041	443 ± 52	0.097	0.451
POISE-WOA	0.845 ± 0.054	0.968 ± 0.023	0.854 ± 0.050	346 ± 132	<0.001	0.586
POISE-HHO	0.865 ± 0.045	0.971 ± 0.018	0.869 ± 0.035	298 ± 155	<0.001	0.312
POISE-EO	0.867 ± 0.044	0.974 ± 0.013	0.873 ± 0.032	226 ± 43	<0.001	0.273
POISE-MPA	0.867 ± 0.043	0.973 ± 0.014	0.871 ± 0.033	311 ± 68	<0.001	0.544
POISE-SMA	0.830 ± 0.050	0.951 ± 0.034	0.843 ± 0.041	80 ± 67	<0.001	0.276
POISE-GBO	0.852 ± 0.059	0.974 ± 0.014	0.864 ± 0.041	264 ± 82	0.001	0.551
POISE-AO	0.882 ± 0.049	0.976 ± 0.021	0.888 ± 0.038	374 ± 161	0.097	2.200
POISE-AOA	0.868 ± 0.048	0.969 ± 0.018	0.872 ± 0.035	343 ± 104	0.007	0.365
POISE-ARO	0.861 ± 0.036	0.971 ± 0.016	0.868 ± 0.029	266 ± 62	0.003	0.523
POISE-GJO	0.840 ± 0.052	0.963 ± 0.013	0.849 ± 0.039	**75 ± 33**	<0.001	0.245
POISE-INFO	0.865 ± 0.047	0.973 ± 0.016	0.872 ± 0.039	381 ± 131	0.001	0.478
POISE-RIME	0.865 ± 0.048	0.973 ± 0.016	0.873 ± 0.039	330 ± 137	0.002	0.617
POISE-SCSO	0.864 ± 0.052	0.968 ± 0.018	0.872 ± 0.041	265 ± 101	<0.001	0.334
POISE-FOX	0.841 ± 0.065	0.951 ± 0.032	0.850 ± 0.058	95 ± 92	<0.001	**0.095**
No selection + LR	0.902 ± 0.050	0.978 ± 0.014	0.902 ± 0.042	2505	–	–
ANOVA top 450 + LR	0.886 ± 0.057	0.973 ± 0.014	0.892 ± 0.045	450	–	–

**Table 4:** No-selection reference: each classifier, and two soft-voting ensembles, trained on all 2505 pooled features of each outer training fold. Mean ± SD over the five outer folds.

Model	Macro-Averaged $F_{1}$	Macro-AUC	Accuracy	$F_{1}$ (*POLE*)
LR	0.902 ± 0.050	0.978 ± 0.014	0.902 ± 0.042	0.897 ± 0.101
SVM + LR (soft vote)	0.894 ± 0.067	0.979 ± 0.015	0.897 ± 0.054	0.877 ± 0.138
All five (soft vote)	0.877 ± 0.047	0.980 ± 0.007	0.881 ± 0.035	0.861 ± 0.124
SVM	0.872 ± 0.049	0.977 ± 0.017	0.883 ± 0.042	0.820 ± 0.098
RF	0.857 ± 0.028	0.975 ± 0.007	0.862 ± 0.030	0.839 ± 0.087
XGBoost	0.851 ± 0.052	0.973 ± 0.015	0.865 ± 0.036	0.789 ± 0.147
KNN	0.789 ± 0.068	0.905 ± 0.044	0.805 ± 0.045	0.721 ± 0.186

**Table 5:** The twenty features most frequently selected by POISE-GWO across its 20 runs. “Selected” is the number of runs in which the feature was in the final panel; “in pool” the number of runs in which it passed the in-fold prefilter. “All 19” is the selection frequency averaged over all 19 instantiations. Copy-number and mRNA features are identified by gene symbol. Fifteen features were selected in eight runs each; 11 of them are listed. Recurrence across runs reflects the behavior of the search on this data set. It does not establish that any listed feature is causally or mechanistically related to a molecular subtype, independently associated with it, or clinically useful, and none of these features should be regarded as a biomarker without external validation.

Feature	Block	Selected	In Pool	All 19
*RPL22*	Mutation	12	16	0.339
*BFSP1*	Mutation	11	20	0.258
cg12790037	Methylation	10	20	0.232
miR-490	miRNA	10	20	0.332
*CACNA1E*	Mutation	10	20	0.308
cg16523137	Methylation	9	20	0.203
miR-1283-1	miRNA	9	20	0.189
*FREM2*	Mutation	9	20	0.168
*MS4A15*	mRNA	9	20	0.205
*SPATA16*	Copy number	8	20	0.182
*SNORA72*	Copy number	8	20	0.174
miR-5088	miRNA	8	20	0.195
miR-5094	miRNA	8	12	0.116
miR-6875	miRNA	8	20	0.218
*AGTPBP1*	Mutation	8	16	0.174
*FRMPD2*	Mutation	8	20	0.218
*GOLGA4*	Mutation	8	20	0.224
*MED12L*	Mutation	8	20	0.295
*TDRD1*	Mutation	8	20	0.229
*HMG20B*	mRNA	8	20	0.200

## Data Availability

The TCGA-UCEC data are available from UCSC Xena (https://xenabrowser.net (accessed on 6 October 2026)) and cBioPortal (https://www.cbioportal.org (accessed on 6 October 2026)). The source code is available at https://github.com/BilanLimch/POISE-X (accessed on 6 October 2026).

## Supplementary Materials

The following supporting information can be downloaded at https://www.mdpi.com/article/10.3390/cancers18203353/s1: Table S1: Leave-one-fold-out evaluation of the configuration-selection rule; Table S2: Single-block, leave-one-block-out, and two-block analyses; Table S3: Comparison with a surrogate of the clinical molecular classification; Table S4: Omics-only and clinical-only analyses; Table S5: Sensitivity to the parsimony weight α; Figure S1: Panel size and macro-averaged $F_{1}$ as functions of α.
