# Supplementary material for: A Leakage-Controlled Benchmark of Metaheuristic Multi-Omics Feature Selection for Reconstructing TCGA Molecular Subtypes in Endometrial Cancer
Source: Cancers (Basel). 2026 Oct 16;18(20):3353. doi: 10.3390/cancers18203353 (PMC13702640; doi:10.3390/cancers18203353)
Supplement: Supplementary file 1 [file cancers-18-03353-s001.zip › cancers-4596472-supplementary.pdf]

Supplementary Materials: A Leakage-Controlled Benchmark of Metaheuristic Multi-Omics Feature Selection for Reconstructing TCGA Molecular Subtypes in Endometrial Cancer

Minyue Cao [1,†], Yiqin Zhang [1,†], Yan Ding [1], Genyi Jiang [1], Jiayu Yan [1], Yihan Sun [1], Yanli Zhang [1], Luyao Kang [1], Jing Luo [1] and Bilan Li [1,*]

This document contains Tables S1–S5 and Figure S1. Section, table, and figure numbers without the prefix S refer to the main text. All the analyses used the outer folds, the in-fold prefilter, and the in-fold standardization of the main experiment (Sections 3.3, 3.5, and 3.6 of the main text).

S1. Evaluation of the Configuration-Selection Rule

The configuration-selection rule of Section 3.8 of the main text was applied in a leave-one-fold-out manner. For each held-out outer fold, the complete rule was applied to the 16 runs of the other four folds: the instantiation with the highest mean outer-test macro-averaged F_1 was taken as the reference, the other 18 instantiations were compared with it by two-sided paired Wilcoxon signed-rank tests with Holm correction, and, among the instantiations not significantly different from the reference—that is, with a Holm-adjusted $p \geq 0.05$—the one with the lowest mean search objective was chosen. The chosen configuration was then scored on the held-out fold as the mean macro-averaged F_1 of its four runs on that fold. Applied to all 20 runs, the same implementation reproduced the Holm-adjusted p-values of Table 3 of the main text and designated POISE-GWO.

Table S1. Leave-one-fold-out evaluation of the configuration-selection rule. For each held-out fold, the rule was applied to the other four folds; POISE-ACO was the reference in every fold. "Group" lists the instantiations not significantly different from the reference; "Objective" is the mean best search objective of the chosen instantiation over those 16 runs. The last three columns give the mean outer-test macro-averaged F_1 over the four runs of the held-out fold. The bottom row gives the mean ± SD over the five folds; "Rule" is the estimate for the selection procedure, and the POISE-ACO column is the estimate for choosing the instantiation with the highest mean score.

Fold	Group	Chosen	Objective	Macro-Averaged F_1 on Held-Out Fold		
				Rule	POISE-ACO	POISE-GWO
0	ACO, AO, DE, GWO, PSO	GWO	0.052	0.929	0.933	0.929
1	ACO, AO, CS, DE, GWO, PSO	GWO	0.054	0.857	0.914	0.857
2	ACO, AO, CS, DE, GWO, PSO	GWO	0.054	0.877	0.893	0.877
3	ACO, AO	AO	0.084	0.809	0.822	0.804
4	ACO, AO, CS, DE, GWO, PSO	GWO	0.053	0.931	0.940	0.931
Mean ± SD				0.881 ± 0.052	0.900 ± 0.047	0.880 ± 0.053

S2. Single-Block and Leave-One-Block-Out Analyses

To assess which data layers are required, LR, the classifier of the no-selection reference, was trained on the 500 in-fold prefiltered features of each omics block alone, and on the full candidate pool with one block left out at a time (Table S2). No single block reached the score of the full pool. The mutation block came closest with a macro-averaged F_1 of 0.817, followed by mRNA with 0.801, methylation with 0.730, miRNA with 0.696, and gene-level copy number with 0.348, which recognized no *POLE* tumor. Leaving out the mutation block caused the largest loss, to 0.838, and the *POLE* F_1 fell from 0.897 to 0.716, whereas leaving out any other block lowered the score by at most 0.028, to values between 0.874

and 0.895. The subtype information was therefore concentrated in, but not confined to, the mutation layer. To identify the smallest combination of layers that approached the full pool, the mutation block was combined with each other omics block and the clinical variables: mutation and methylation data together reached 0.897 with 1005 features, within 0.005 of the full pool, whereas mutation with mRNA, miRNA, or copy number reached 0.880, 0.843, and 0.833.

Table S2. Outer-test performance of LR trained on single omics blocks, on the candidate pool with one block left out, and on the mutation block combined with one other omics block. Mean ± SD over the five outer folds. The full-pool row is the no-selection reference of Table 3 of the main text; the leave-one-block-out and two-block pools keep the five clinical variables.

Input	Features	Macro-Averaged F_1	Macro-AUC	F_1 (*POLE*)
Single block				
mRNA	500	0.801 ± 0.070	0.950 ± 0.025	0.614 ± 0.223
miRNA	500	0.696 ± 0.062	0.881 ± 0.038	0.500 ± 0.133
Methylation	500	0.730 ± 0.040	0.933 ± 0.015	0.486 ± 0.101
Copy number	500	0.348 ± 0.024	0.699 ± 0.039	0.000 ± 0.000
Mutation	500	0.817 ± 0.080	0.955 ± 0.022	0.860 ± 0.180
Leave one block out				
Without mRNA	2005	0.889 ± 0.065	0.976 ± 0.016	0.862 ± 0.142
Without miRNA	2005	0.895 ± 0.054	0.979 ± 0.015	0.907 ± 0.097
Without methylation	2005	0.874 ± 0.039	0.971 ± 0.019	0.859 ± 0.116
Without copy number	2005	0.890 ± 0.047	0.977 ± 0.015	0.871 ± 0.124
Without mutation	2005	0.838 ± 0.047	0.959 ± 0.023	0.716 ± 0.159
Mutation and one other block				
Mutation + mRNA	1005	0.880 ± 0.062	0.976 ± 0.015	0.871 ± 0.124
Mutation + miRNA	1005	0.843 ± 0.076	0.961 ± 0.021	0.855 ± 0.108
Mutation + methylation	1005	0.897 ± 0.056	0.978 ± 0.016	0.907 ± 0.097
Mutation + copy number	1005	0.833 ± 0.085	0.962 ± 0.017	0.870 ± 0.184
Full pool	2505	0.902 ± 0.050	0.978 ± 0.014	0.897 ± 0.101

S3. Comparison with a Surrogate of the Clinical Molecular Classification

In routine care, the four molecular groups are assigned by *POLE* exonuclease-domain sequencing and immunohistochemistry for mismatch-repair proteins and p53 [1]. As a comparator, three indicators that mirror these assays were derived from the same TCGA data without using the subtype labels: (i) *POLE*: one of the 11 pathogenic exonuclease-domain variants listed by León-Castillo et al. [2], namely the five hotspot mutations P286R, V411L, S297F, A456P, and S459F and the six further pathogenic variants F367S, L424I, M295R, P436R, M444K, and D368Y, carried by 37 patients. (ii) Mismatch-repair deficiency: a non-silent mutation of *MLH1*, *MSH2*, *MSH6*, or *PMS2*, found in 81 patients, or *MLH1* promoter hypermethylation, found in 91 patients, 145 patients in total; promoter hypermethylation was defined as a mean beta value above 0.3 over the 27 probes of the 450K array that lie within 500 bp of the *MLH1* transcription start site (NM_000249.4, hg38), a threshold that falls in the trough of the clearly bimodal distribution of this mean. (iii) *TP53*: a non-silent *TP53* mutation, found in 143 patients. The indicators were combined either by LR trained on the three indicators in each outer training fold or by a sequential rule that followed the current molecular classification [3] and assigned *POLE*-mutated tumors first, then mismatch-repair-deficient tumors, then *TP53*-mutated tumors, and all remaining tumors to the group with no specific molecular profile; these four groups were mapped to the TCGA classes *POLE*-ultramutated, MSI-hypermutated, copy-number-high, and copy-number-low, respectively. This order differs from that of the original ProMisE algorithm, which tests mismatch repair first [1], and it matters in these data: 35 of the 37 patients with a pathogenic

POLE variant also carried a non-silent mutation in a mismatch-repair gene, as expected for ultramutated tumors, and 34 of these 35 belonged to the *POLE*-ultramutated class.

The rule had no parameters fitted to the subtype labels and assigned 338 of the 369 patients to their TCGA class, a proportion of 0.916. Most disagreements were 17 copy-number-high tumors without a *TP53* mutation that the rule placed in the copy-number-low group and 6 copy-number-low tumors with a *TP53* mutation placed in the copy-number-high group. LR trained on the three indicators obtained the same outer-test scores as the rule in every fold. With a macro-averaged F_1 of 0.924, the surrogate scored higher than every multi-omics model of this study, including LR on the full candidate pool with 0.902 (Table S3). The advantage lay in the class assignments and came from the *POLE* class, 0.962 against 0.897, and the MSI class, 0.968 against 0.913. The macro-AUC of the LR surrogate was lower than that of the full-pool model, 0.959 against 0.978, and so was the F_1 of the copy-number-high class, 0.893 against 0.915, and of the copy-number-low class, 0.874 against 0.881. With *MLH1* methylation thresholds of 0.2 and 0.4 instead of 0.3, the rule reached a macro-averaged F_1 of 0.919 ± 0.030 and 0.922 ± 0.027. A higher score of the surrogate was expected, because the TCGA classes were themselves defined largely by these genomic properties [4].

Table S3. Outer-test performance of the surrogate of the clinical molecular classification compared with the multi-omics models. Mean ± SD over the five outer folds for the surrogate and the no-selection reference, and over the 20 runs for POISE-GWO (Table 3 of the main text). The sequential rule outputs a single class and has no class probabilities, so no AUC is reported for it.

Model	Features	Macro-Averaged F_1	Macro-AUC	Accuracy	F_1 (*POLE*)
Surrogate, sequential rule	3	0.924 ± 0.030	–	0.916 ± 0.031	0.962 ± 0.056
Surrogate, LR	3	0.924 ± 0.030	0.959 ± 0.016	0.916 ± 0.031	0.962 ± 0.056
No selection + LR	2505	0.902 ± 0.050	0.978 ± 0.014	0.902 ± 0.042	0.897 ± 0.101
POISE-GWO	443 ± 52	0.880 ± 0.054	0.975 ± 0.012	0.884 ± 0.041	0.862 ± 0.129

S4. Omics-Only Sensitivity Analysis

Because histology is strongly associated with the copy-number-high subtype, the main analyses were repeated without the five clinical variables, so that the candidate pool contained the 2500 omics features only. The no-selection reference, the ANOVA-ranked panel of 450 features, and the searches of POISE-GWO and POISE-ACO were repeated on this pool with 20 runs each and the seeds of the main experiment, and LR was also trained on the five clinical variables alone (Table S4). Removing the clinical variables changed the macro-averaged F_1 of LR on the full pool from 0.902 to 0.896, that of POISE-GWO from 0.880 to 0.875, and that of POISE-ACO from 0.900 to 0.894. The ANOVA-ranked panel of 450 features was unchanged at 0.886, because no clinical variable ranked among the top 450 features in any fold; the best-ranked clinical variable, endometrioid histology, ranked 876th or lower. In paired comparisons with the main experiment, which used the same folds and seeds, neither search differed significantly in a two-sided Wilcoxon signed-rank test, with $p = 0.812$ for POISE-GWO and $p = 0.099$ for POISE-ACO; as in Table 3 of the main text, these tests were exploratory. The F_1 of the copy-number-high class, the class most strongly associated with serous histology, was 0.903 for POISE-GWO and 0.914 for POISE-ACO without the clinical variables, against 0.908 and 0.923 with them. LR on

the five clinical variables alone reached a macro-averaged F_1 of 0.378. The classification performance reported in the main text therefore did not depend on the clinical variables.

Table S4. Outer-test performance with and without the five clinical variables. Mean ± SD over the five outer folds for the LR models and over the 20 runs for the searches. The rows with clinical variables are those of Table 3 of the main text; $|S|$ is the number of features used.

Model	$\|S\|$	Macro-Averaged F_1	Macro-AUC
With clinical variables (main experiment; 2505 candidate features)			
No selection + LR	2505	0.902 ± 0.050	0.978 ± 0.014
ANOVA top 450 + LR	450	0.886 ± 0.057	0.973 ± 0.014
POISE-GWO	443 ± 52	0.880 ± 0.054	0.975 ± 0.012
POISE-ACO	1193 ± 22	0.900 ± 0.046	0.979 ± 0.013
Omics only (2500 candidate features)			
No selection + LR	2500	0.896 ± 0.060	0.978 ± 0.014
ANOVA top 450 + LR	450	0.886 ± 0.057	0.973 ± 0.014
POISE-GWO	430 ± 77	0.875 ± 0.049	0.976 ± 0.012
POISE-ACO	1177 ± 22	0.894 ± 0.044	0.978 ± 0.013
Clinical variables only			
LR	5	0.378 ± 0.029	0.725 ± 0.021

S5. Sensitivity to the Parsimony Weight

The searches of POISE-GWO and POISE-ACO were run with $\alpha \in \{0.8, 0.9, 0.95, 0.99\}$ in Equation (1) of the main text, with the outer folds, seeds, population size, and evaluation budget of the main experiment and with 20 runs per instantiation and a value of α; $\alpha = 0.9$ was the main experiment (Table S5 and Figure S1). Panel sizes changed moderately with α, from 346 to 479 features for POISE-GWO and from 1123 to 1228 features for POISE-ACO between $\alpha = 0.8$ and $\alpha = 0.99$, and the gap between the compact and the large-panel search persisted at every value; the difference in panel size between the two instantiations was therefore driven by their search dynamics, not by the penalty weight. The macro-averaged F_1 ranged from 0.869 to 0.881 for POISE-GWO and from 0.883 to 0.900 for POISE-ACO, and no configuration exceeded the no-selection reference of 0.902 at any value of α. POISE-ACO obtained its highest score at $\alpha = 0.9$; at $\alpha = 0.8$ and $\alpha = 0.99$ its fold mean was lower than at $\alpha = 0.9$ in all five folds. At the other three values of α, POISE-ACO exceeded POISE-GWO by 0.006–0.014, less than the 0.020 at $\alpha = 0.9$, which is a further reason not to read the ranking of individual optimizers too precisely.

Table S5. Outer-test performance and panel size as functions of the parsimony weight α. Mean ± SD over 20 runs. "Objective" is the mean best value of Equation (1) reached by the searches; values are not comparable across α. The rows for $\alpha = 0.9$ are those of Table 3 of the main text. The no-selection reference with LR was 0.902 ± 0.050.

Instantiation	α	Macro-Averaged F_1	Macro-AUC	$\|S\|$	Objective
POISE-GWO	0.8	0.869 ± 0.047	0.974 ± 0.017	346 ± 53	0.058
	0.9	0.880 ± 0.054	0.975 ± 0.012	443 ± 52	0.053
	0.95	0.881 ± 0.048	0.975 ± 0.016	470 ± 81	0.049
	0.99	0.878 ± 0.036	0.977 ± 0.013	479 ± 77	0.043
POISE-ACO	0.8	0.883 ± 0.050	0.978 ± 0.013	1123 ± 30	0.132
	0.9	0.900 ± 0.046	0.979 ± 0.013	1193 ± 22	0.094
	0.95	0.891 ± 0.047	0.979 ± 0.011	1204 ± 35	0.073
	0.99	0.884 ± 0.044	0.977 ± 0.014	1228 ± 26	0.055

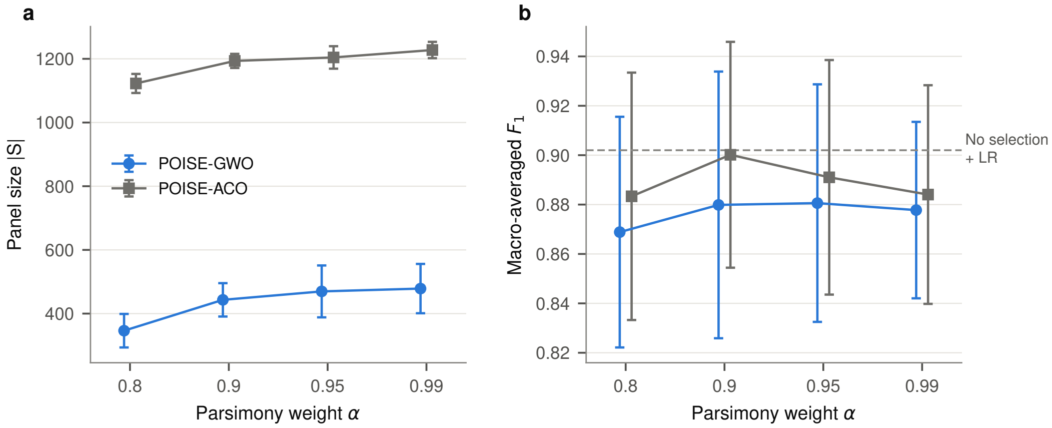

Figure S1. Panel size (**a**) and outer-test macro-averaged F_1 (**b**) of POISE-GWO and POISE-ACO as functions of the parsimony weight α (mean ± SD over 20 runs). The dashed line in (**b**) is the no-selection reference with LR.

References

1. Talhouk, A.; McConechy, M.; Leung, S.; Li-Chang, H.; Kwon, J.; Melnyk, N.; Yang, W.; Senz, J.; Boyd, N.; Karnezis, A.; et al. A clinically applicable molecular-based classification for endometrial cancers. *Br. J. Cancer* **2015**, *113*, 299–310. https://doi.org/10.1038/bjc.2015.190.
2. León-Castillo, A.; Britton, H.; McConechy, M.; McAlpine, J.; Nout, R.; Kommoss, S.; Brucker, S.; Carlson, J.; Epstein, E.; Rau, T.; et al. Interpretation of somatic POLE mutations in endometrial carcinoma. *J. Pathol.* **2020**, *250*, 323–335. https://doi.org/10.1002/path.5372.
3. Concin, N.; Matias-Guiu, X.; Vergote, I.; Cibula, D.; Mirza, M.; Marnitz, S.; Ledermann, J.; Bosse, T.; Chargari, C.; Fagotti, A.; et al. ESGO/ESTRO/ESP guidelines for the management of patients with endometrial carcinoma. *Int. J. Gynecol. Cancer* **2021**, *31*, 12–39. https://doi.org/10.1136/ijgc-2020-002230.
4. Levine, D.; The Cancer Genome Atlas Research Network. Integrated genomic characterization of endometrial carcinoma. *Nature* **2013**, *497*, 67–73. https://doi.org/10.1038/nature12113.
